# The Valuable Prognostic Impact of Regional Lymph Node Removed on Outcomes for IIIA N0 NSCLC Patients

**DOI:** 10.7150/jca.86495

**Published:** 2023-07-09

**Authors:** Yukun Wang, Zixuan Liu, Zhonghua Zhou, Hanyu Rao, Jie Xiong, Shuanshuan Xie

**Affiliations:** 1 Department of Respiratory Medicine, Shanghai Tenth People's Hospital, Tongji University; Shanghai 200040, China.; 2 Tongji University School of Medicine; Shanghai 200072, China.; 3 Department of Respiratory Medicine, ChongMing Branch of Shanghai Tenth People's Hospital, Tongji University School of Medicine, Shanghai 202150, China.

**Keywords:** regional lymph nodes, non-small cell lung cancer, lung cancer-specific survival, overall survival

## Abstract

**Background:** Regional lymph nodes (RLNs) removed combined with surgery is a standard option for patients at stage I to IIIA NSCLC. The objective of the study is to clarify the effect of removing different number of RLNs on survival outcomes for patients at stage IIIA N0 NSCLC.

**Methods:** Patients at stage IIIA N0 NSCLC from 2004 to 2015 were identified from Surveillance, Epidemiology, and End Results (SEER) database. Prior propensity score method (PSM), survival time was compared among different number (0, 1-3 and ≥4) of RLNs removed groups. After PSM, lung cancer-specific survival (LCSS) and overall survival (OS) were compared. Kaplan-Meier analysis and Cox regression analyses were used to clarify the impact of the factors on the prognosis with hazard ratio (HR) and 95% confidence interval (CI).

**Results:** A total of 11,583 patients at stage IIIA N0 NSCLC were included. Prior PSM, survival indicators including 1-year mortality rate, 5-year mortality rate, median survival time (MDST) and mean survival time (MST) from good to bad were all: ≥4, 1-3 and none RLNs removed group. After PSM, Kaplan-Meier survival analyses and univariate Cox regression analyses on OS and LCSS revealed a statistically significance on survival curve (*P*<0.001) between each two of the three groups (none, 1-3 and ≥4 RLNs removed group). Multivariable Cox regression analyses on OS and LCSS showed an independent association of RLNs removed with higher OS (HR, 0.275; 95% CI, 0.259-0.291; *P*<0.001) and LCSS (HR, 0.239; 95% CI, 0.224-0.256; *P*<0.001) compared with none RLN removed and no statistical difference with OS (HR, 1.118; 95% CI, 0.983-1.271; *P*=0.088) and LCSS (HR, 1.107; 95% CI, 0.954-1.284; *P*=0.179) between 1-3 RLNs removed and ≥4 RLNs removed.

**Conclusions:** Removing RLNs was beneficial to survival outcomes of patients at stage IIIA N0 NSCLC. Compared with 1-3 RLNs removed, ≥4 RLNs removed could bring a better survival time but not an independent prognostic factor (*P*>0.05).

## 1. Introduction

Lung cancer (LC) is the leading cause of cancer-related death 1. In the latest global cancer statistics, LC is with high incidence and high mortality characteristics which is 11.4% and 18.0% of all cancers, ranking the first and second, respectively 2. 1,796,144 people in 185 countries died of LC in 2020, indicating that the research about LC is of great significance. NSCLC is associated with the characteristics of high morbidity and mortality in all lung malignancy subtypes. Approximately, 85% of lung cancer are NSCLC and the 5-year survival rate for NSCLC patients is about 15-20% 3, 4. However, only about one-third of NSCLC patients who were diagnosed early may be cured by resection of tumor 5. As the epidemic of NSCLC, it is concerned by people and has become a serious global public health problem.

According to National Comprehensive Cancer Network Guidelines (Version 1.2020, 2019) in the US, suitable patients at stage I to IIIA NSCLC may be recommended to have a surgery to cure which may be the best way to cure NSCLC. During the surgery, RLNs is a standard treatment for patients with NSCLC 6. However, the optimal number of RLNs during surgery is still controversial for the IIIA NSCLC patients. The ACOSOG Z0030 trial suggested the optimal number of resection lymph node (LN) should be 10 7. The American College of Surgeons' Commission on Cancer considered a removal of at least 10 regional LNs might be adequate 8. Currently, the Union for International Cancer Control and American Joint Committee on Cancer both endorsed 6 LNs for resection 9.

At present, there are few clinic studies on the impact of the RLNs removed on the survival of NSCLC patients, especially for IIIA stage. Our study is aimed to clarify the optimal number of RLNs removed for IIIA N0 NSCLC, and finger out the prognostic factors for them.

## 2. Materials and Methods

### 2.1. Data source

We conducted this retrospective study to clarify the impact of the number of RLNs removed on patients at stage IIIA N0 NSCLC. The data were retrieved from SEER database, which was established by the National Cancer Institute in the United States (US) in 1973. The SEER database keeps providing incidence, survival, and mortality data for histopathologic cancer subtypes and data by molecular subtyping, covering approximately 30% population of the US 10.

### 2.2. Study population

**Figure 1:**
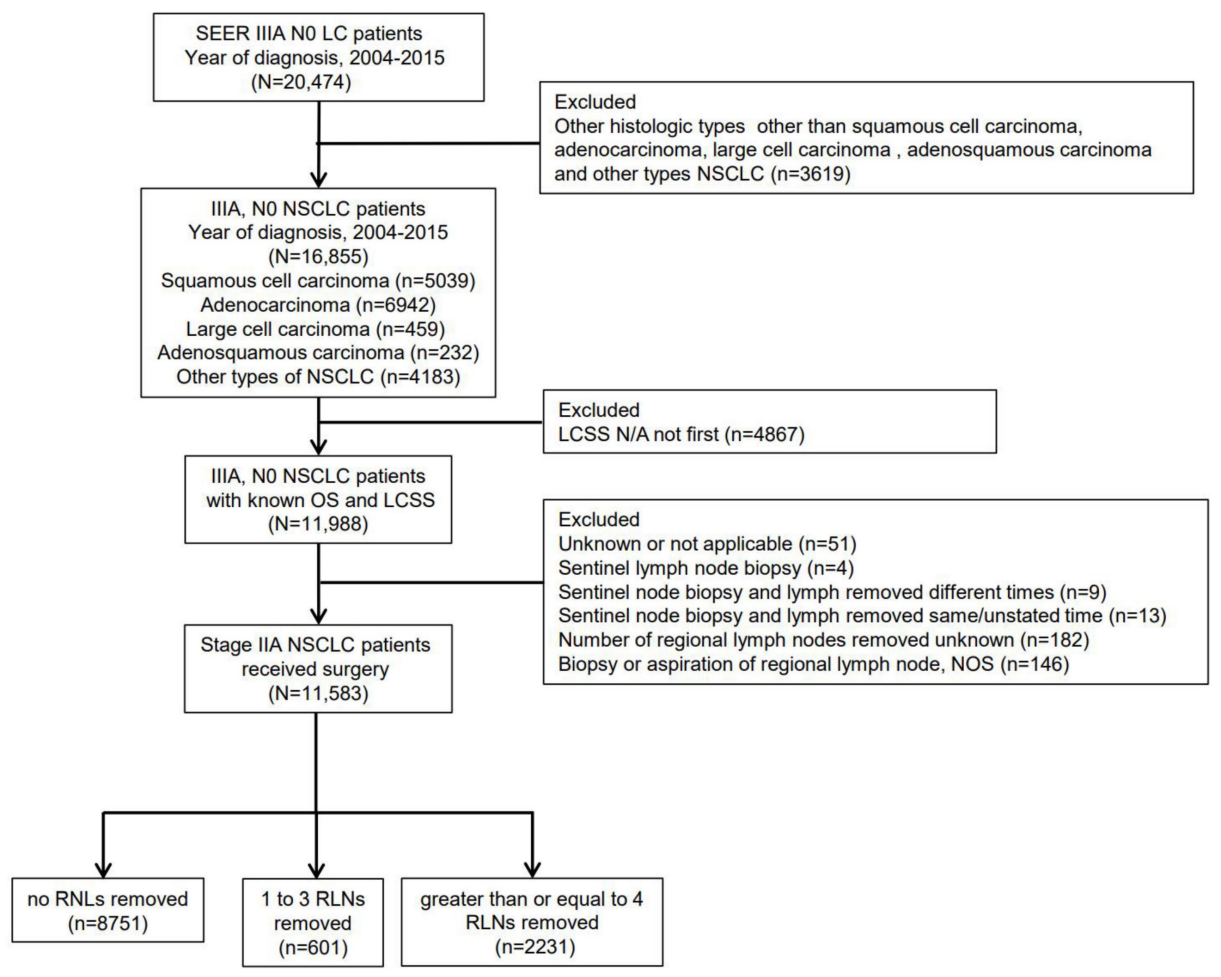
Study flow diagram. SEER, Surveillance, Epidemiology and End Results; LC, lung cancer; NSCLC, non-small cell lung cancer; N/A, not applicable; OS, overall survival; LCSS, lung cancer-specific survival; NOS, not otherwise specified; RLN, regional lymph node.

Eligible LC patients were identified initially from SEER database between 2004 and 2015. We limited the cohort to NSCLC patients diagnosed with adenocarcinoma (pathological codes 8140/3), squamous cell carcinoma (pathological codes 8070/3), adenosquamous carcinoma (pathological codes 8560/3), large cell carcinoma (pathological codes 8012/3) and other types of NSCLC. The patients with not applicable LCSS were excluded. The number of RLNs removed was divided into three categories: none RLN removed group, 1-3 RLNs removed group and ≥4 RLNs removed group. Detailed criterion is shown in Figure 1. “Excluded LCSS N/A not first” meant the research has ruled out that the tumors studied were not the first tumors to occur.

### 2.3. Covariates

Baseline clinical characteristics including age, survival time, size of tumor, gender, race, region, year of diagnosis, primary site, grade, laterality, pathology of tumor, stage, radiation record (RT), chemotherapy record (CT), radiation sequence with surgery, insurance, high school education (%), marital status, median household income (US dollars, tens) and number of RLNs removed were collected.

### 2.4. Statistical analyses

Potential deviation between none RLN removed group and RLNs group, 1-3 RLNs removed and ≥4 RLNs removed group were controlled by PSM analysis (1:3). The survival curves were created by using Kaplan-Meier analysis with log-rank test to compare OS and LCSS for the various RLNs removed categories, RT record and CT record among the cohort after PSM. Univariate and multivariate Cox regression analyses were used to clarify the impact and independence of predictors to survival outcomes: OS and LCSS. Predictors (*P*<0.05) identified in Kaplan-Meier analyses or univariable analyses were included into multivariable analysis.

Continuous variables were compared by using t-test, and categorical variables were compared by using chi-square. Data were analyzed using IBM SPSS version 21.0 (IBM Corp, Armonk, NY, USA). The forest plots were generated by GraphPad Prism (version 8.0, GraphPad Software Inc, San Diego, CA, USA). Statistical significance was set at a two-tailed *P*<0.05.

## 3. Results

### 3.1. Study cohort characteristics

Figure 1 shows a flow diagram of the study for the detailed criterion about patients from the SEER database. A total of 11,583 patients at stage IIIA T4, M0, N0 NSCLC were included among the cohort, with the histological type of squamous cell carcinoma (n=3730), adenocarcinoma (n=4720), adenosquamous carcinoma (n=148), large cell carcinoma (n=335) and other types of NSCLC (n=2650). These 11,583 patients were divided into three categories: 8751 patients without any RLN removed group (75.5%), 601 patients with 1-3 RLNs removed group (5.2%) and 2231 patients with ≥4 RLNs removed group (19.3%).

We compared the clinical, histological, social, demographic and therapeutic characteristics between none RLN removed group and RLNs removed group, 1-3 RLNs removed and ≥4 RLNs removed group both prior and after PSM (Table 1,2). Prior PSM, compared with none RLN removed group, the proportions from not first to first were age between 65 to 74 (38.1%), diagnosis year between 2012-2015 (34.0%) and poorly differentiated (36.3%) in RLNs removed group. Besides, female (47.8%), white race (84.7%), tumor size ≤1 cm (96.4%), tumor on upper lobe (62.9%), laterality right-origin of primary (57.9%), adenocarcinoma (43.3%), no CT record (59.6%), insured (53.5%) married (56.2%) and income >5000, ≤7000 (50.7%) were more common in RLNs removed group. Compared with 1-3 RLNs removed group, the proportions from not first to first were diagnosis year between 2012-2015 (35.5%) and pacific coast region (43.2%) in ≥4 RLNs removed group. Besides, age between 55 to 64 (26.7), male (52.8%), tumor size ≤1 cm (97.0%), tumor on upper lobe (63.3%), poorly differentiated (36.8%), laterality right-origin of primary (58.0%), adenocarcinoma (44.1%), no CT record (59.8%), insured (55.2%) married (57.2%) and income >5000, ≤7000 (51.5%) were more common in ≥4 RLNs removed group. The largest to small proportion of applications of RT were: none RLNs removed group, RLNs removed group and 1-3 RLNs removed group and ≥4 RLNs removed group.

After PSM, there were 8496 patients without any RLN removed and 2832 patients with RLNs removed. Among the cohort of RLNs removed, there were 601 patients with 1-3 RLNs removed and 1803 patients with ≥4 RLNs removed.

### 3.2. Survival outcomes

The 1-year mortality rate was 51.8% (5998 of 11583), including 5543 deaths (63.3%), 123 deaths (20.5%) and 332 deaths (14.8%) in the none RLN removed group, 1-3 RLNs removed group and ≥4 RLNs removed group (*P*<0.05). 5-year survival rate, MDST and MST prior PSM were shown in Table 3. All survival indicators from good to bad were: ≥4 RLNs removed group, 1-3 RLNs removed group and none RLN removed group.

**Figure 2:**
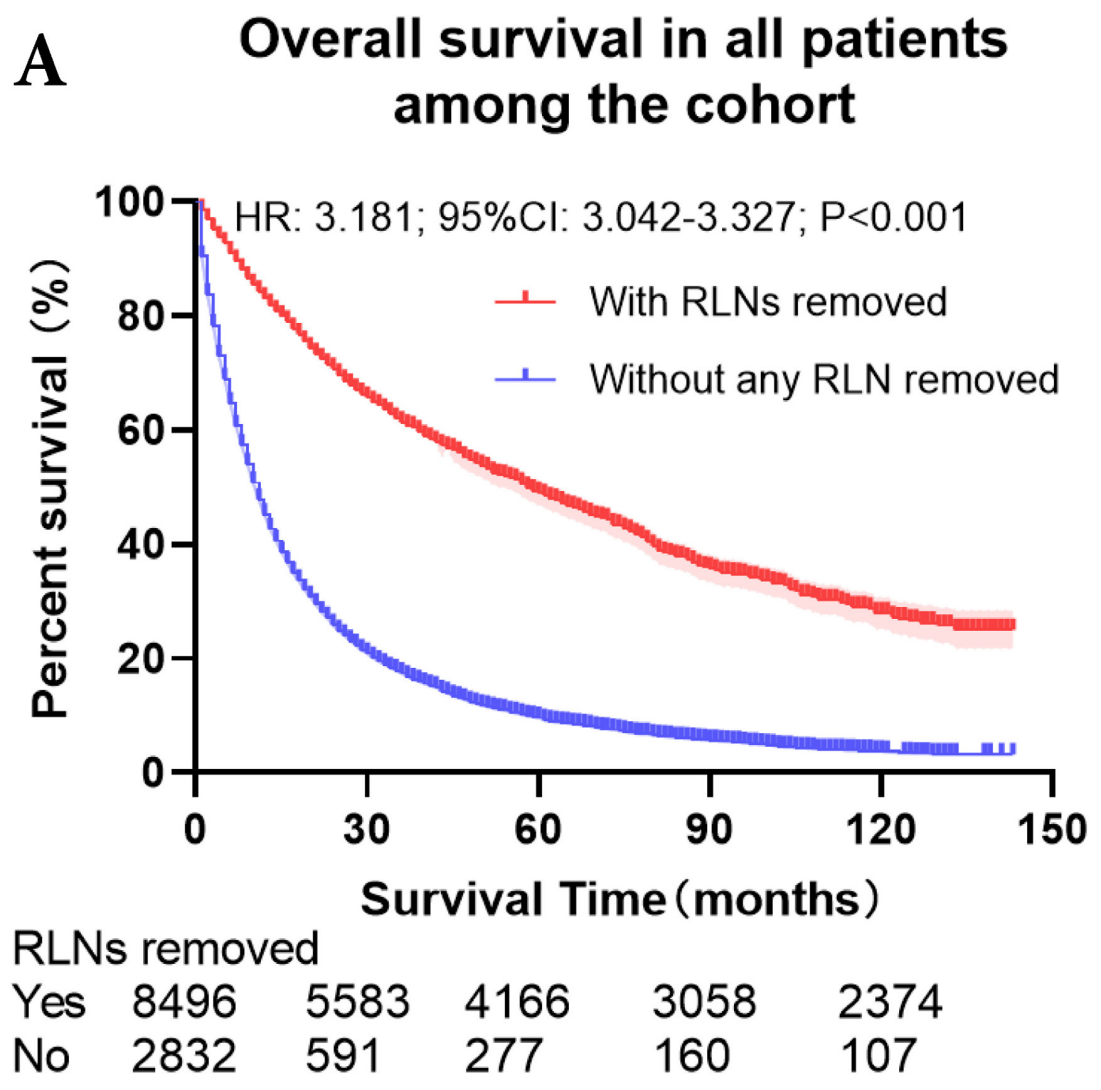

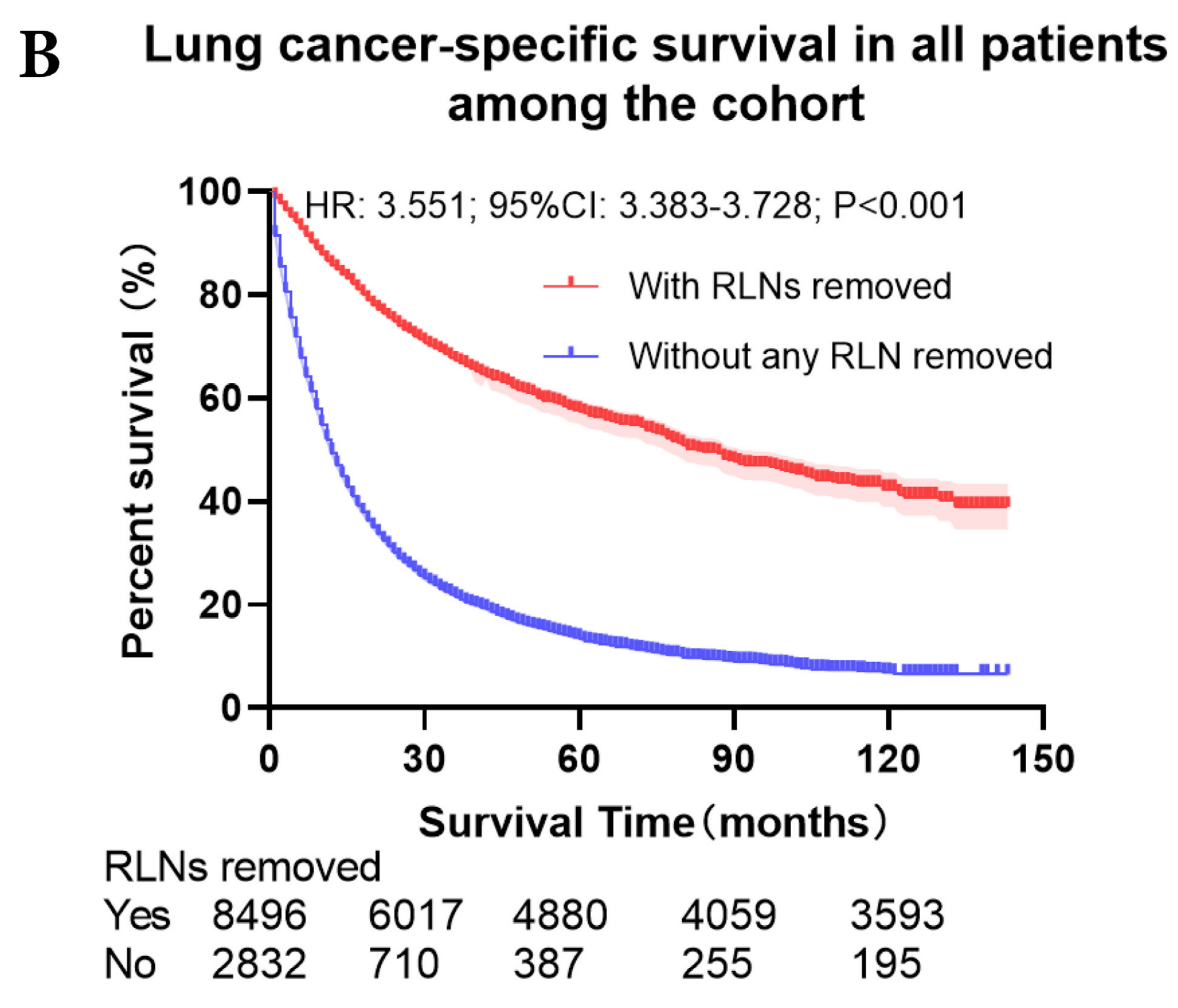
Comparison of survival curve: role of RLNs removed on survival outcome: (**a**) RLNs removed that can influence OS; (**b**) LNs removed that can influence LCSS. RLN, regional lymph node; HR, hazard ratio; CI, confidence interval.

**Figure 3:**
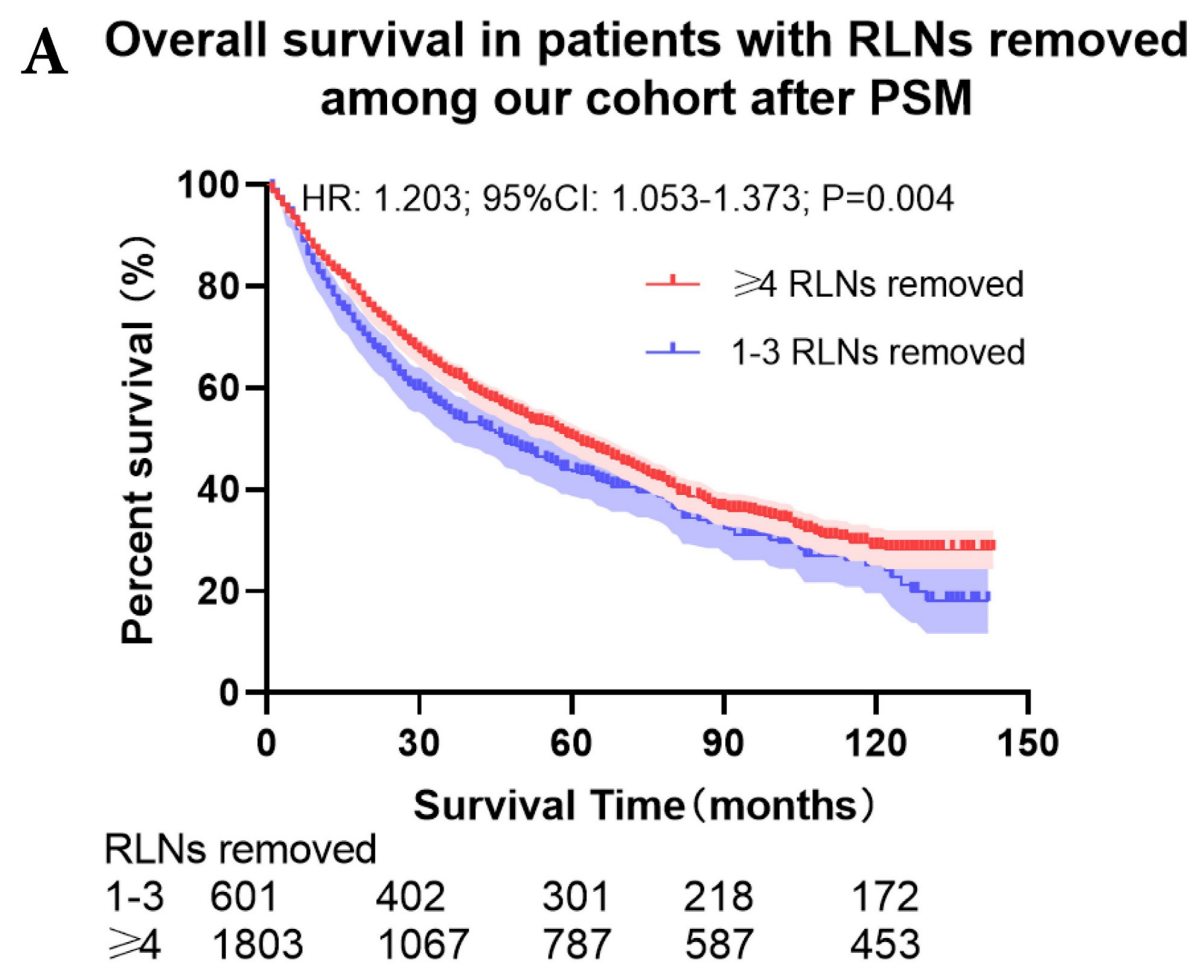

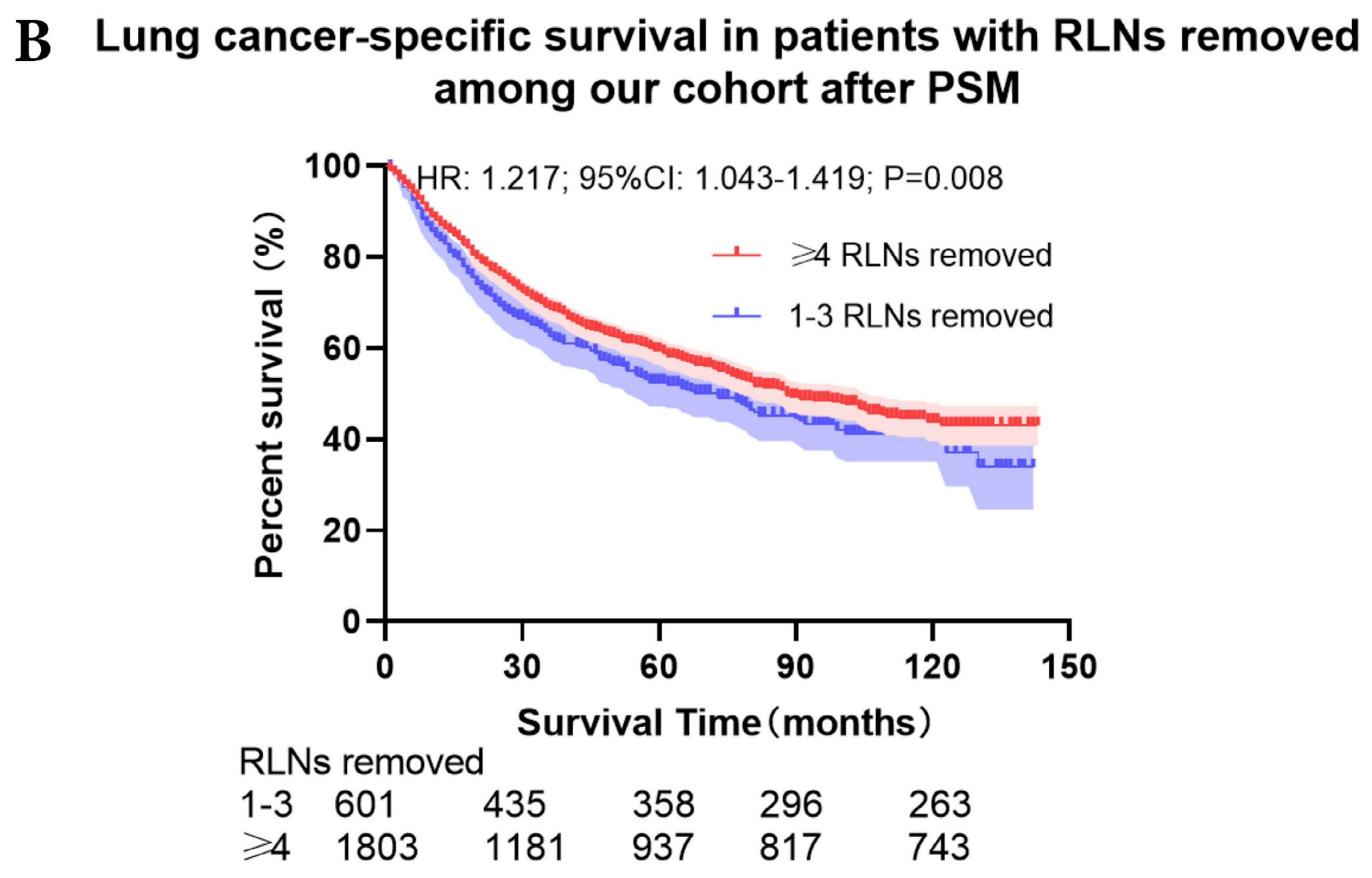
Comparison of survival curve: role of different number of RLNs removed on survival outcome: (**a**) Different number of RLNs removed that can influence OS; (**b**) Different number of RLNs removed that can influence LCSS. RLN, regional lymph node; HR, hazard ratio; CI, confidence interval.

The survival curves showed that there were statistically significances both on OS and LCSS among the cohort after PSM with regard to number of RLNs removed (*P*<0.001 and *P*<0.001, respectively), CT (*P*<0.001 and *P*<0.001 with curves crossing, respectively) and RT (*P*<0.001 with curves crossing and *P*<0.001 with curves crossing, respectively) between without and with RLNs removed group by using Kaplan-Meier analysis with log-rank test (Figure 2, Figure S1). Between 1-3 RLNs removed group and ≥4 RLNs removed group, the survival curves showed that there were also statistically significances both on OS and LCSS among the cohort after PSM with regard to number of RLNs removed (*P*=0.002 and *P*=0.004, respectively) and RT (*P*<0.001 with curves crossing and *P*<0.001 with curves crossing, respectively) by using Kaplan-Meier analysis with log-rank test (Figure 3, Figure S2A, S2B). In terms of CT, there were statistically significant differences on LCSS among the cohort after PSM with regard to (*P*=0.008 with curves crossing) except OS (*P*=0.833) between 1-3 RLNs removed group and ≥4 RLNs removed group (Figure S2C, S2D).

**Figure 4:**
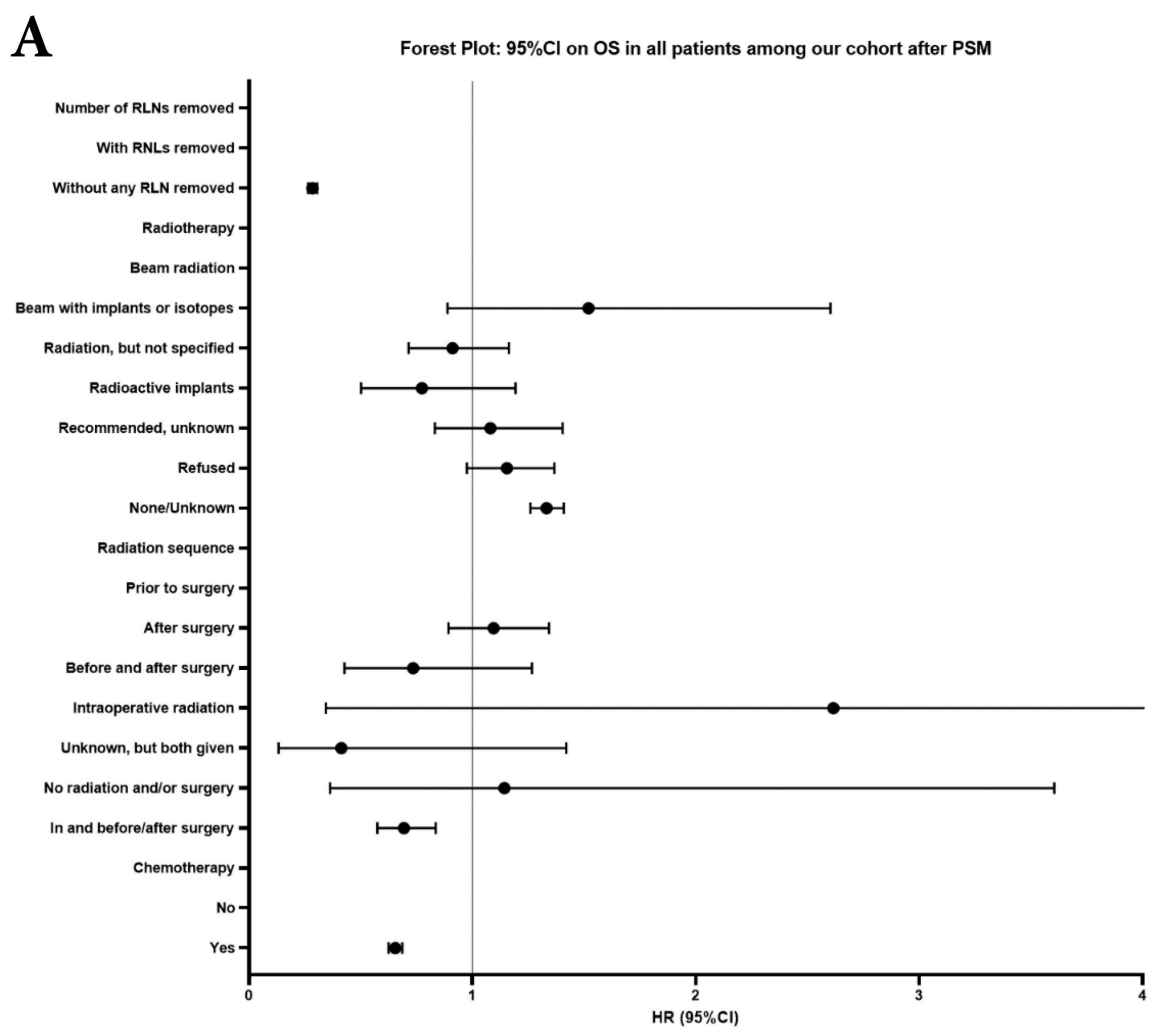

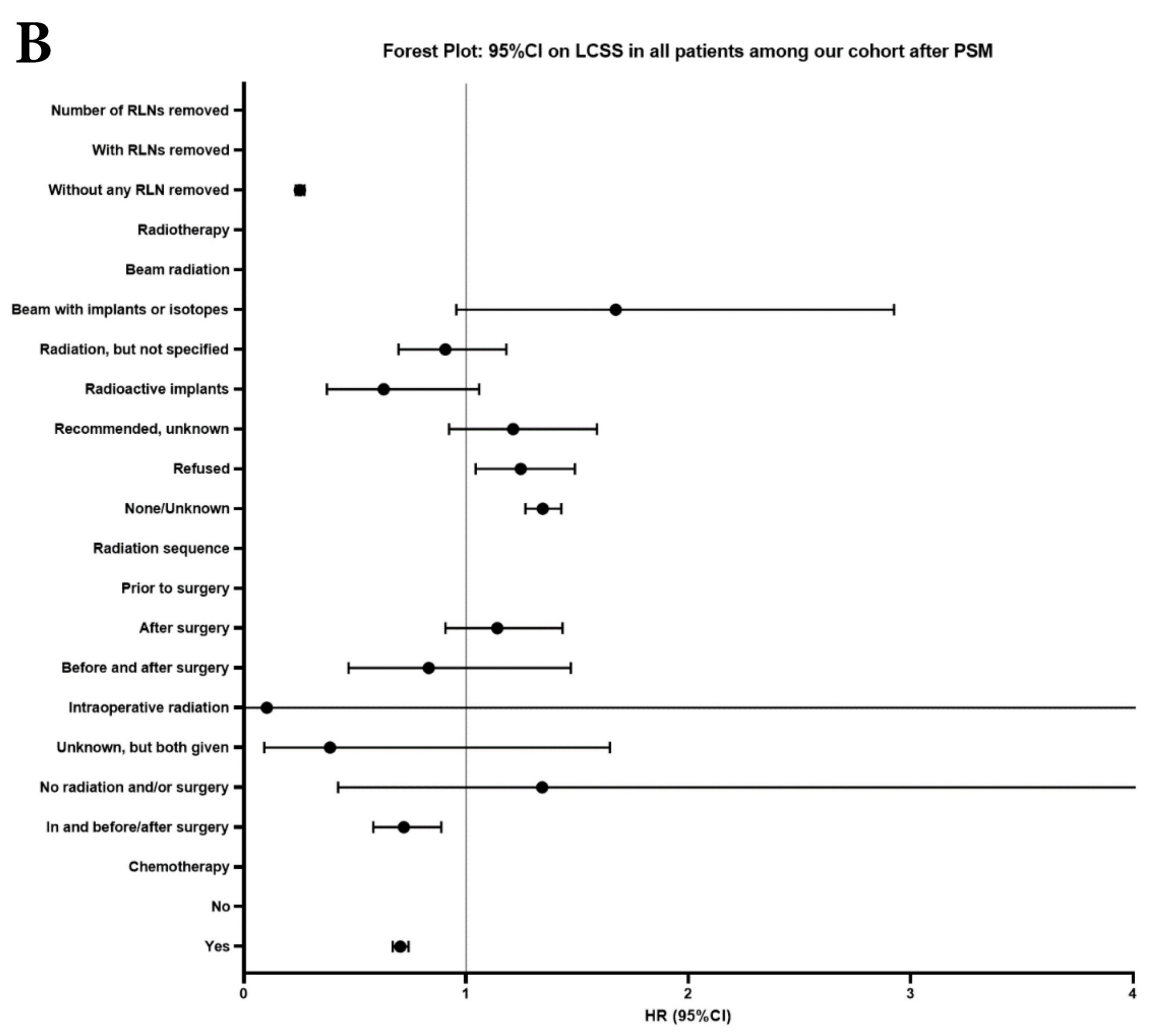

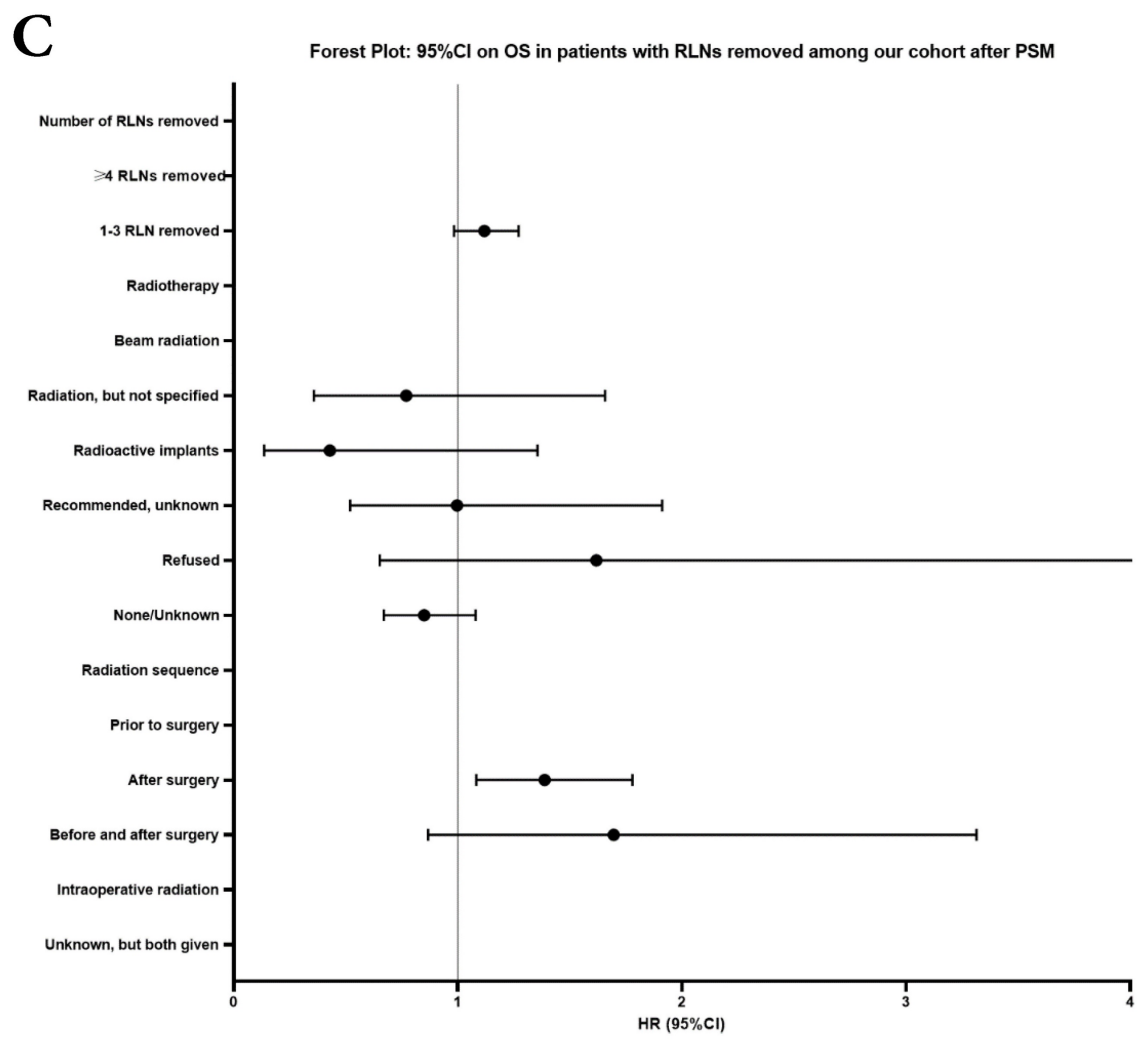

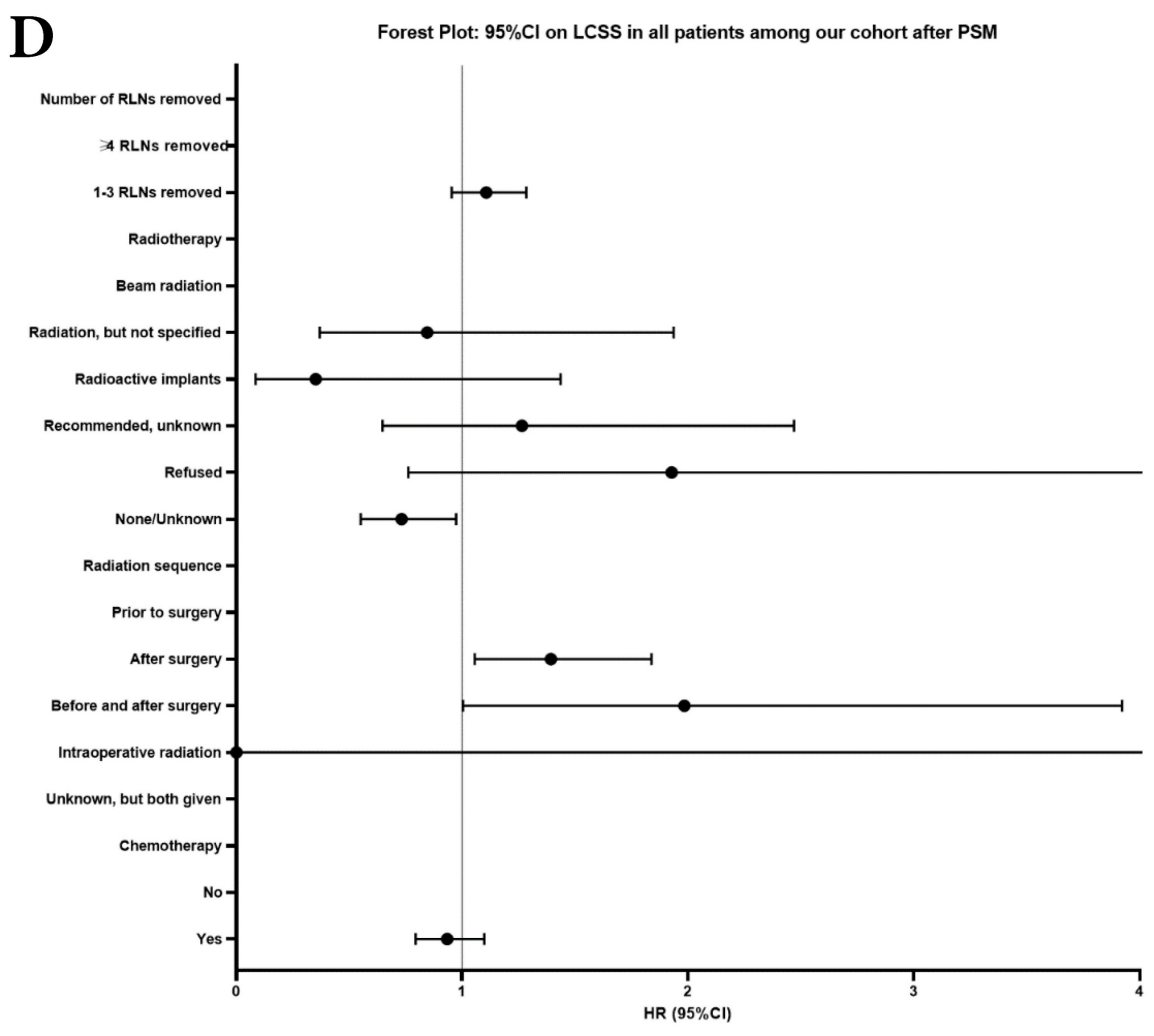
Forest plot of HRs of factors that can influence OS and LCSS in patients by multivariable Cox regression: (**a**) HRs of factors that can influence OS in all patients among our cohort; (**b**) HRs of factors that can influence LCSS in all patients among our cohort; (**c**) HRs of factors that can influence OS in patients with RLNs removed among our cohort; (**d**) HRs of factors that can influence LCSS in patients with RLNs removed among our cohort.

Univariate and multivariable analysis on OS and LCSS between without and with RLNs removed group, 1-3 RLNs removed group and ≥4 RLNs removed group were shown in Table 4,5. Multivariable Cox regression analysis revealed independent associations of RLNs removed with higher OS (HR, 0.275; 95% CI, 0.259-0.291; *P*<0.001) and LCSS (HR, 0.239; 95% CI, 0.224-0.256; *P*<0.001) compared with none RLNs removed (Table 4). Furthermore, a smaller number of RLNs removed (1-3 RLNs) was found to be no statistical difference with OS (HR, 1.118; 95% CI, 0.983-1.271; *P*=0.088) and LCSS (HR, 1.107; 95% CI, 0.954-1.284; *P*=0.179) compared with a larger number of RLNs removed (≥4 RLNs) (Table 5). In addition, the forest plots of HRs for OS were generated to show the same multivariable Cox regression analysis outcomes of treatments which is also factors that can be changed even when the patient have been suffered from NSCLC. between without and with RLNs removed group, 1-3 RLNs removed group and ≥4 RLNs removed group more visually (Figure 4).

## 4. Discussion

Surgical treatment with RLNs removed is the mostly common applied treatment of NSCLC patients especially for stage I-IIIA 6, 11. Although the research about the number of RLNs are keeping increasing these years, there is still no specific recommendation for the number of LN removed in any guideline. Only few articles are about this topic, whose conclusions are still controversial. Dai et al. found that for gradually elevated T stage (mainly stage I), examination of more and more LNs seems to be crucial for survival outcomes 12. David et al. concluded that compared with the ≥10 LNs removed, <10 LNs removed was associated with poor overall survival for stage I NSCLC patients 13. Liang et al. reported that 16 examined LNs could be the cut point for prognostic stratification postoperatively for NSCLC patients with declared node-negative disease 14. Cao et al. reported that LN dissection, especially more extensive RLN removed (≥4 RLNs) is associated with a higher survival rate in patients at stage IA NSCLC tumors ≤2 cm underwent sublobar resection 15. The accuracy of staging is often affected by the number of LNs examined and different stages correspond to different treatment and different prognosis 12. So, the exploration of the optimal number of LN removed needs to be studied after classify different stage of NSCLC. National Comprehensive Cancer Network Guidelines (Version 1.2020, 2019) only recommended suitable patients at stage IIIA NSCLC may be considered to have a surgery to cure which may be the only way to cure NSCLC, but there was no recommendation on the number of LNs removed. Unfortunately, there are few studies specifically evaluated the survival benefits of removing different number of RLNs for patients with IIIA NSCLC.

In our research, we found that there was better prognosis in RLNs removed group compared with none RLN removed group. In terms of 1-3 RLNs removed group and ≥4 RLNs removed group, survival indicators (1-year mortality rate, 5-year survival rate, MDST and MST), Kalan-Meier survival analyses and univariate Cox regression analyses on OS and LCSS all revealed that there was a better prognosis for ≥4 RLNs removed group. But, multivariable Cox regression analyses on OS and LCSS showed there was no statistical difference between two groups above (Figure 4). Here we found an interesting conclusion that under the premise of RLNs removed, the number of RLNs removed was not an independent prognostic factor, but ≥4 RLNs removed can indeed improve the prognosis. We can conclude that RLNs removed is an important treatment for stage IIIA N0 NSCLCN patients, and ≥4 RLNs removed seems to bring a better survival time but not an independent prognostic factor, compared with 1-3 RLNs removed. Besides, when we focused on the cohort only including 1-3 RLNs removed group and ≥4 RLNs removed group, the independent prognostic factors (age, sex, tumor location, histologic type and insurance) became much less compared with the cohort including with and without RLNs removed group, indicating that unchangeable factors (age, sex, tumor location and histologic type) are more important for patients choosing RLNs removed. In addition to paying more attention to the insurance situation, which can be changed, manual intervention cannot bring better effects, which maybe it will bring harm, considering the inconsistent effects of CT and RT in survival analysis (further discussion below).

It is well known that surgery with CT is an optimal treatment for stage IA-IIIA NSCLC patients 11, 16. Even aggressive consolidative therapy may appear to improve survival in patients with persistent or high nodal burden disease 17. Ito et al. reported that there was a significant difference in the OS and disease-free survival rates in the intralobar group which is opposite in the and hilar group according to adjuvant CT 18. In our research, we found that whether in the whole cohort or in patients with RLNs removed, less patients chose CT than did not and the proportion of CT applied was reduced on the premise of RLN removed and CT was a controversial factor for the prognosis. Only between without and with RLNs removed group, CT is definitely good for prognosis on OS. Between 1-3 RLNs removed group and ≥4 RLNs removed group, there were no statistically significance of CT on OS. In terms of LCSS, the effects CT between without and with RLNs removed group, 1-3 RLNs removed group and ≥4 RLNs removed group seem to be indeterminate, whose survival curves crossing, demonstrating that CT has no clear benefits, and may also bring harm instead, under the premise of RLNs removed. The National Comprehensive Cancer Network guidelines (Version 3.2023) recommended conventionally fractionated RT for locally advanced IIIA N0 NSCLC. The role of RT in the treatment seems more non-uniform. RT may yield a negative effect on survival outcomes in surgical patients with IIA NSCLC, which is consistent with the National Comprehensive Cancer Network guidelines (Version 1.2020, 2019), which recommends CT, but not RT, for patients at stage IIA NSCLC 19. In terms of stage IIIA NSCLC, the conclusions are still controversial. Shinde et al. concluded that there was no benefit observed for adjuvant CT or RT in the entire cohort which include patients with non-metastatic, cN2 (IIIA or IIIB) NSCLC diagnosed from 2004 to 2015 17. However, Liu et al. reported that patients can benefit from adjuvant RT if they have more than 5 positive RLNs, larger tumors (≥3cm), and older age (≥65 years old) 20. In our research, whether in the whole cohort or in patients with RLNs removed, less patients chose RT than did not and the proportion of RT applied was keeping reducing with the number of RLNs removed increasing. And, all survival curves in terms of RT crossed, demonstrating that RT has no clear benefits, and may bring harm instead, under the premise of RLNs removed. The same as exploration of the number of LN removed, the explorations of CT or RT need be done based on detailed classification, considering that even if there is only a very small difference in stage, the treatment will be different or even the opposite.

The latest edition of the International Union Against Cancer TNM staging standard for lung cancer was announced in January 2017 9. The 8th TNM classification changed definitions in terms of tumor size, which provides a higher level of differentiation based on global database, extensive internal validation, sophisticated analyses and multiple evaluations that confirm generalizability 19. Ashwin et al. found that the therapeutic effect of CT and RT was different in different LN ratio which meat number of nodes involved by tumor divided by number of nodes examined of LNs, highlighting the importance of LN biopsy and new TNM classification 17. LN biopsy is important for staging, which is important for the choice of treatment, and the 8th TNM classification makes a more accurate and personalized treatment opportunity for stage IIIA NSCLC patients, considering that it provides a standard for the scope of LN removed. At present, the significance of LN removed in radical surgery of NSCLC mainly includes two aspects: one is to ensure complete tumor resection, which directly reduce the probability of recurrence; another is biopsy, ensuring the accuracy of LN staging so as to help develop a better treatment plan for patients, that is, for prognosis 21, 22. Studies have found that even if the tumor is completely removed in the early stage of the disease, a considerable number of patients still have the risk of recurrence, suggesting that it is related to LN micrometastasis, and once again confirmed the importance of RLNs removed. In this study, our findings are consistent with the opinions above, which may provide a theoretical basis for the necessity of RLNs removed.

**Figure 5:**
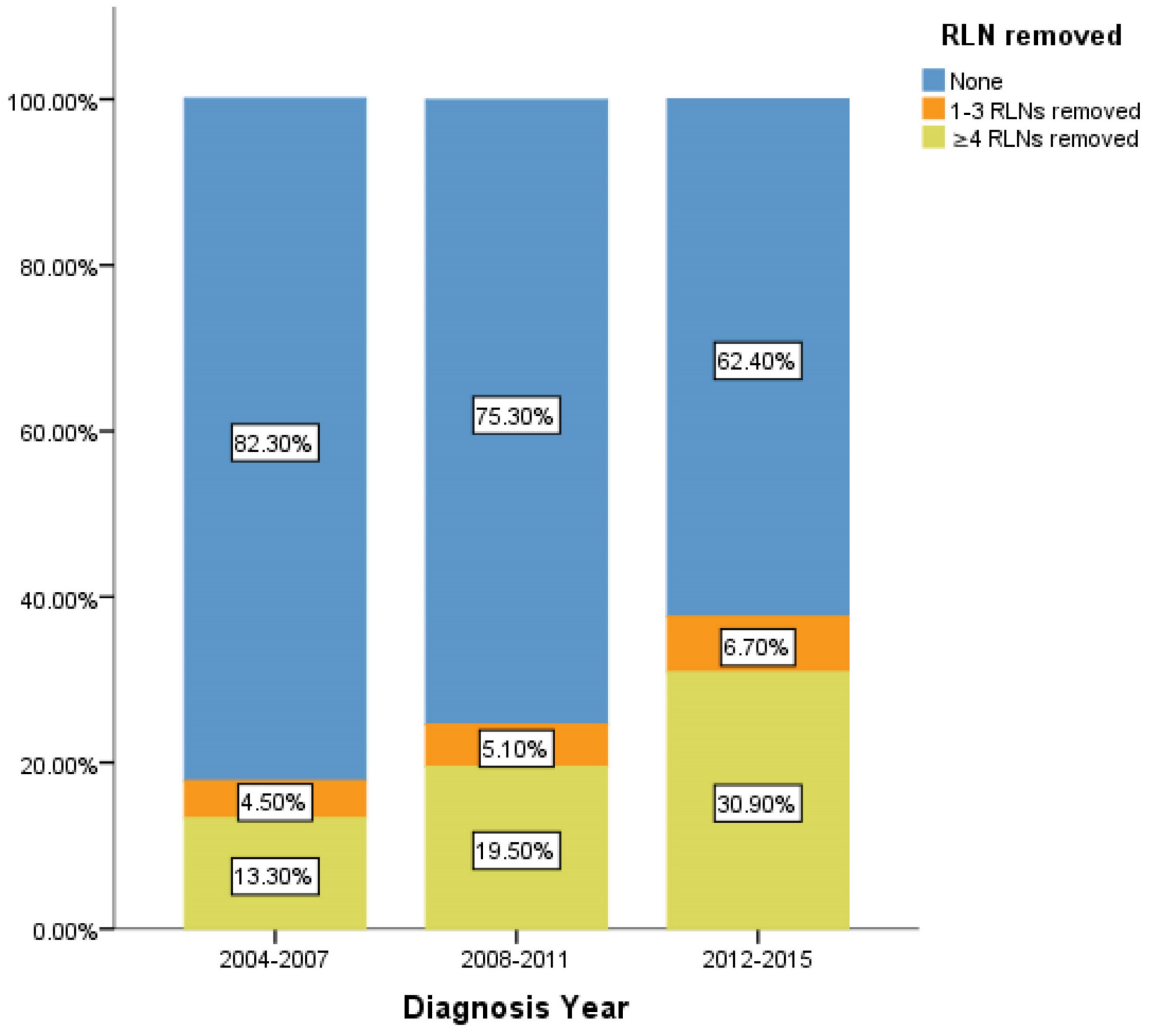
Percentage of IIIA N0 NSCLC patients of different number of RLNs removed over time (2004-2015). RLN, regional lymph node.

However, there is still limitation in our research. The data of RLNs are limited by the inherent flaw of the SEER database, which did not record specific number of RLNs removed. Our research found compared with 1-3 RLNs removed, ≥4 RLNs removed brings a better survival time but not an independent prognostic factor. But it is well known that infinite removal of RLNs cannot be sustained conducive to prognosis, on the contrary, there may be side effects. The topic of what specific number RLNs removed specifically are enough and best seems to be important 23. Limited by the fact that the SEER database does not provide specific numbers, we cannot determine specific cut point number of RLNs in ≥4RLNs group. This information could be included in further research. However, our research has reported the positive effect of RLNs removed for the prognosis based on 11,583 patients and 17 variables among our cohort. All the comparisons were made after PSM which eliminated the baseline difference basically. Therefore, in the background of absent large-scale data from prospective trials and clinical guideline, our conclusion not only is highly reliable, but also brings a conclusion which could provide accurate reference about RLNs removed treatment for the patients at stage IIIA N0 NSCLC. We also found that 24.45% of patients at stage IIIA N0 NSCLC had RLNs removed, 5.2% and 19.26% of them had 1-3 RLNs and ≥4 RLNs removed, respectively between 2004 to 2015. In recent 12 years, there is a sustained growth trend for the number of RLNs removed (Figure 5). The proportion of 1-3 RLNs removed and ≥4 RLNs removed group was increased from 4.5% to 6.7% and 13.3% to 30.9%, respectively, dedicating that more and more clinicians are aware of the benefits of having more RLNs removed. The phenomenon mentioned above also was consistent with the finding that diagnosis year was an independent prognostic factor and the later the diagnosis year, the better the prognosis. 6.7% and 30.9% indicates the clinical significance that such benefit option still needs to be applied for the better prognosis of IIIA N0 NSCLC patients, which dedicated the significance of our conclusion again. Considering an interesting conclusion that under the premise of RLNs removed, the number of RLNs removed was not an independent prognostic factor, but ≥4 RLNs removed can indeed improve the prognosis, we predicted that the number of RLNs removed and diagnosis year were associated variables. This prediction will be researched in future research.

## 5. Conclusions

Removing RLNs was beneficial to survival outcomes of patients at stage IIIA N0 NSCLC. Compared with 1-3 RLNs removed, ≥4 RLNs removed brought a better survival time but not an independent prognostic factor (*P*>0.05).

## Supplementary Material

Supplementary figures.

### Author Contributions

Conceptualization, S.S.X. and Y.K.W.; methodology, S.S.X. and Y.K.W.; software, Y.K.W., Z.X.L. and H.Y.R.; validation, Z.H.Z.; formal analysis, Z.H.Z.; investigation, N/A; resources, S.S.X.; data curation, S.S.X. and Y.K.W.; writing—original draft preparation, Y.K.W. and Z.X.L.; writing—review and editing, Z.H.Z., Y.K.W. and S.S.X.; visualization, Y.K.W.; supervision, S.S.X.; project administration, S.S.X.; funding acquisition, S.S.X. All authors have read and agreed to the published version of the manuscript.

### Funding

This research was funded by National Natural Science Foundation of China, grant number 82272673.

### Informed Consent Statement

Patient consent was waived due to this article is from SEER database, which is publicly available deidentified patients' data from National Cancer Institute (NCI), USA.

### Data Availability Statement

All data generated during this study are included in this article. The datasets supporting the conclusions of this article are available in SEER database: https://seer.cancer.gov/, accessed on 20 July 2022.

## Figures and Tables

**Table 1:** Baseline characteristics of patients at stage IIIA N0 NSCLC with and without RLNs removed prior and after PSM

Variable	Full cohort				Matched cohort		
	None RLN removed	With RLNs removed	*P* value		None RLN removed	With RLNs removed	*P* value
Age			<0.001				<0.001
≤45	132 (1.5)	52 (1.8)			130 (1.5)	52 (1.8)	
45-54	644 (7.4)	270 (9.5)			625 (7.4)	270 (9.5)	
55-64	1641 (18.8)	736 (26.0)			1585 (18.7)	736 (26.0)	
65-74	2668 (30.5)	1079 (38.1)			2573 (30.3)	1079 (38.1)	
≥75	3666 (41.9)	695 (24.5)			3583 (42.4)	695 (24.5)	
Sex			0.007				0.019
Female	3929 (44.9)	1354 (47.8)			4650 (54.7)	1478 (52.2)	
Male	4822 (55.1)	1478 (52.2)			3846 (45.3)	1354 (47.8)	
Race			<0.001				<0.001
White	6916 (79.0)	2398 (84.7)			6719 (79.1)	2399 (84.7)	
Black	1182 (13.5)	260 (9.2)			1142 (13.4)	260 (9.2)	
Others	647 (7.4)	168 (5.9)			629 (7.4)	168 (5.9)	
Unknown	6 (0.1)	6 (0.2)			6 (0.1)	6 (0.2)	
Tumor Size			<0.001				<0.001
≤1 cm	5986 (68.4)	2731 (96.4)			5967 (70.2)	2731 (96.4)	
>1, ≤2 cm	3 (0.0)	2 (0.1)			3 (0.0)	2 (0.1)	
>2, ≤3 cm	7 (0.1)	2 (0.1)			7 (0.1)	2 (0.1)	
>3, ≤4 cm	7 (0.1)	3 (0.1)			7 (0.1)	3 (0.1)	
>4 cm	2 (0.0)	0 (0)			2 (0.0)	0 (0.0)	
Unknown	2746 (31.4)	94 (3.3)			2510 (29.5)	94 (3.3)	
Diagnosis year			<0.001				<0.001
2004-2007	4232 (48.4)	931 (32.2)			4061 (47.8)	913 (32.2)	
2008-2011	2916 (33.3)	955 (33.7)			2849 (33.5)	955 (33.7)	
2012-2015	1603 (18.3)	964 (34.0)			1586 (18.7)	964 (34.0)	
Tumor location			<0.001				<0.001
Main bronchus	558 (6.4)	53 (1.9)			484 (5.7)	42 (1.5)	
Upper lobe	4401 (50.3)	1782 (62.9)			4333 (51.0)	1782 (62.9)	
Middle lobe	288 (3.3)	89 (3.1)			280 (3.3)	89 (3.1)	
Lower lobe	1944 (22.2)	809 (28.6)			1913 (22.5)	809 (28.6)	
Overlapping lesion	110 (1.3)	57 (2.0)			110 (1.3)	57 (2.0)	
Not otherwise specified	1450 (16.6)	42 (1.5)			1376 (16.2)	53 (1.9)	
Differentiation grade			<0.001				<0.001
Well differentiated	309 (3.5)	387 (13.7)			304 (3.6)	387 (13.7)	
Moderately differentiated	1365 (15.6)	1018 (35.9)			1342 (15.8)	1018 (35.9)	
Poorly differentiated	2391 (27.3)	1028 (36.3)			2364 (27.8)	1028 (36.3)	
Undifferentiated	146 (1.7)	51 (1.8)			145 (1.7)	51 (1.8)	
Unknown	4540 (51.9)	348 (12.3)			4341 (51.1)	348 (12.3)	
Laterality			<0.001				<0.001
Left-origin of primary	3882 (44.4)	1189 (42.0)			3759 (44.2)	1189 (42.0)	
Right-origin of primary	4678 (53.5)	1639 (57.9)			4565 (53.7)	1639 (57.9)	
One side, unspecified	32 (0.4)	2 (0.1)			3 (0.4)	2 (0.1)	
Paired site	147 (1.7)	2 (0.1)			136 (1.6)	2 (0.1)	
Not a paired site	12 (0.1)	0 (0.0)			6 (0.1)	0 (0.0)	
Histologic type			<0.001				<0.001
Adenocarcinoma	3490 (39.9)	1230 (43.4)			3422 (40.3)	1230 (43.4)	
Squamous cell carcinoma	2908 (33.2)	822 (29.0)			2780 (32.7)	822 (29.0)	
Adenosquamous	83 (0.9)	65 (2.3)			82 (1.0)	65 (2.3)	
Large cell carcinoma	264 (3.0)	71 (2.5)			255 (3.0)	71 (2.5)	
Other types of NSCLC	2006 (22.9)	644 (22.7)			1957 (23.0)	644 (22.7)	
Radiotherapy record			<0.001				<0.001
Beam radiation	3597 (41.1)	620 (21.9)			3354 (39.5)	620 (21.9)	
Beam with implants or isotopes	21 (0.2)	2 (0.1)			13 (0.2)	2 (0.1)	
Radiation, but not specified	75 (0.9)	12 (0.4)			73 (0.9)	12 (0.4)	
Radioactive implants	24 (0.3)	7 (0.2)			22 (0.3)	7 (0.2)	
Recommended, unknown	64 (0.7)	21 (0.7)			64 (0.8)	21 (0.7)	
Refused	158 (1.8)	7 (0.2)			158 (1.9)	7 (0.2)	
None/Unknown	4812 (55.0)	2163 (76.4)			4812 (56.6)	2163 (76.4)	
Radiation sequence			<0.001				<0.001
Prior to surgery	26 (0.3)	185 (6.5)			26 (0.3)	185 (6.5)	
After surgery	124 (1.4)	433 (15.3)			124 (1.5)	433 (15.3)	
Before and after surgery	7 (0.1)	19 (0.7)			7 (0.1)	19 (0.7)	
Intraoperative radiation	5 (0.1)	0 (0)			0 (0.0)	1 (0.0)	
Unknown, but both given	1 (0.0)	3 (01)			4 (0.0)	0 (0.0)	
No radiation and/or surgery	8588 (98.1)	2191 (77.4)			8334 (98.1)	2191 (77.4)	
In and before/after surgery	0 (0)	1 (0.0)			1 (0.0)	3 (0.1)	
Chemotherapy record			0.001				0.006
Yes	3835 (43.8)	1143 (40.4)			3680 (43.3)	1143 (40.4)	
No	4916 (56.2)	1689 (59.6)			4816 (56.7)	1689 (59.6)	
Marital status			<0.001				<0.001
Married	4056 (46.3)	1591 (56.2)			3952 (46.5)	1591 (56.2)	
Separated	94 (1.1)	27 (1.0)			91 (1.1)	27 (1.0)	
Single	1210 (13.8)	340 (12.0)			1170 (13.8)	340 (12.0)	
Divorced	1024 (11.7)	366 (12.9)			987 (11.6)	366 (12.9)	
Unmarried or domestic partner	5 (0.1)	2 (0.1)			5 (0.1)	2 (0.1)	
Widowed	2054 (23.5)	410 (14.5)			1991 (23.4)	410 (14.5)	
Unknown	308 (3.5)	96 (3.4)			300 (3.5)	96 (3.4)	
Median family income (dollar, tens)			<0.001				<0.001
≤5000	2801 (32.0)	724 (25.6)			2273 (32.1)	724 (25.6)	
>5000, ≤7000	4105 (46.9)	1435 (50.7)			3965 (46.7)	1435 (50.7)	
>7000, ≤9000	1568 (17.9)	579 (20.4)			1537 (18.1)	579 (20.4)	
>9000	277 (3.2)	94 (3.3)			271 (3.2)	94 (3.3)	

**Abbreviations:** NSCLC, non-small cell lung cancer; RLN, regional lymph node.

**Table 2:** Baseline characteristics of patients at stage IIIA N0 NSCLC with and without RLNs removed prior and after PSM

Variable	Full cohort				Matched cohort		
	1-3 RLNs removed	≥4 RLNs removed	*P* value		1-3 RLNs removed	≥4 RLNs removed	*P* value
Age			0.591				0.782
≤45	11 (1.8)	41 (1.8)			11 (1.8)	32 (1.8)	
45-54	58 (9.7)	212 (9.5)			58 (9.7)	163 (9.0)	
55-64	141 (23.5)	594 (26.7)			141 (23.5)	471 (26.1)	
65-74	234 (38.9)	845 (37.9)			234 (38.9)	676 (37.5)	
≥75	157 (26.1)	538 (24.1)			157 (26.1)	461 (25.6)	
Sex			0.244				0.604
Female	300 (49.9)	1054 (47.2)			301 (50.1)	925 (51.3)	
Male	301 (50.1)	1177 (52.8)			300 (49.9)	878 (48.7)	
Race			0.888				0.951
White	511 (85.0)	1887 (84.6)			511 (85.0)	1533 (85.0)	
Black	57 (9.5)	203 (9.1)			57 (9.5)	162 (9.0)	
Others	32 (5.3)	136 (6.1)			32 (5.3)	104 (5.8)	
Unknown	1 (0.2)	5 (0.2)			1 (0.2)	4 (0.2)	
Tumor Size			0.009				0.059
≤1 cm	567 (94.3)	2164 (97.0)			567 (94.3)	1740 (96.5)	
>1, ≤2 cm	0 (0.0)	2 (0.1)			0 (0.0)	2 (0.1)	
>2, ≤3 cm	1 (0.2)	1 (0.0)			1 (0.2)	1 (0.1)	
>3, ≤4 cm	0 (0.0)	3 (0.1)			0 (0.0)	3 (0.2)	
>4 cm	0 (0.0)	0 (0.0)			0 (0.0)	0 (0.0)	
Unknown	33 (5.5)	61 (2.7)			33 (5.5)	57 (3.2)	
Diagnosis year			<0.001				0.123
2004-2007	231 (38.4)	682 (30.6)			231 (38.4)	614 (34.1)	
2008-2011	199 (33.1)	756 (33.9)			199 (33.1)	617 (34.2)	
2012-2015	171 (28.5)	793 (35.5)			171 (28.5)	572 (31.7)	
Tumor location			0.001				0.046
Main bronchus	370 (61.6)	1412 (63.3)			13 (2.2)	27 (1.5)	
Upper lobe	20 (3.3)	69 (3.1)			370 (61.6)	1136 (63.0)	
Middle lobe	168 (28.0)	641 (28.7)			20 (28.0)	61 (3.4)	
Lower lobe	23 (3.8)	30 (1.3)			168 (28.0)	530 (29.4)	
Overlapping lesion	7 (1.2)	50 (2.2)			7 (1.2)	19 (1.1)	
Not otherwise specified	13 (2.2)	29 (1.3)			23 (3.8)	30 (1.7)	
Differentiation grade			0.005				0.161
Well differentiated	80 (13.3)	307 (13.8)			80 (13.3)	262 (14.5)	
Moderately differentiated	202 (33.6)	816 (36.6)			202 (33.6)	638 (35.4)	
Poorly differentiated	206 (34.3)	822 (36.8)			206 (34.3)	637 (35.3)	
Undifferentiated	12 (2.0)	39 (1.7)			12 (2.0)	37 (2.1)	
Unknown	101 (16.8)	247 (11.1)			101 (16.8)	229 (12.7)	
Laterality			0.046				0.048
Left-origin of primary	346 (57.6)	1293 (58.0)			253 (42.1)	750 (41.6)	
Right-origin of primary	253 (42.1)	936 (42.0)			346 (57.6)	1053 (58.4)	
One side, unspecified	0 (0.0)	2 (0.1)			0 (0.0)	0 (0.0)	
Paired site	2 (0.3)	0 (0.0)			2 (0.3)	0 (0.0)	
Not a paired site	0 (0.0)	0 (0.0)			0 (0.0)	0 (0.0)	
Histologic type			0.031				0.555
Adenocarcinoma	247 (41.1)	983 (44.1)			247 (41.1)	799 (44.3)	
Squamous cell carcinoma	162 (27.0)	660 (29.6)			162 (27.0)	479 (26.6)	
Adenosquamous	20 (3.3)	45 (2.0)			20 (3.3)	45 (2.5)	
Large cell carcinoma	13 (2.2)	58 (2.6)			13 (2.2)	40 (2.2)	
Other types of NSCLC	159 (26.5)	485 (21.7)			159 (26.5)	440 (24.4)	
Radiotherapy record			<0.001				0.016
Beam radiation	177 (29.5)	443 (19.9)			177 (29.5)	410 (22.7)	
Beam with implants or isotopes	0 (0.0)	2 (0.1)			3 (0.5)	0 (0.0)	
Radiation, but not specified	3 (0.5)	9 (0.4)			0 (0.0)	8 (0.4)	
Radioactive implants	3 (0.5)	4 (0.2)			3 (0.5)	4 (0.2)	
Recommended, unknown	4 (0.7)	17 (0.8)			4 (0.7)	13 (0.7)	
Refused	3 (0.5)	4 (0.2)			3 (0.5)	4 (0.2)	
None/Unknown	411 (68.4)	1752 (78.5)			411 (68.4)	1364 (75.7)	
Radiation sequence			<0.001				<0.001
Prior to surgery	36 (6.0)	149 (6.7)			36 (6.0)	120 (6.7)	
After surgery	140 (23.3)	293 (13.1)			140 (23.3)	288 (16.0)	
Before and after surgery	4 (0.7)	15 (0.7)			4 (0.7)	13 (0.7)	
Intraoperative radiation	1 (0.2)	0 (0.0)			1 (0.2)	0 (0.0)	
Unknown, but both given	2 (0.3)	1 (0.0)			2 (0.3)	1 (0.1)	
No radiation and/or surgery	418 (69.6)	1773 (79.5)			418 (69.6)	1381 (76.6)	
In and before/after surgery	1 (0.2)	0 (0.0)			0 (0.0)	0 (0.0)	
Chemotherapy record			0.748				0.399
Yes	246 (40.9)	897 (40.2)			246 (40.9)	703 (39.0)	
No	355 (59.1)	1334 (59.8)			355 (59.1)	1100 (61.0)	
Marital status			0.060				0.702
Married	314 (52.2)	1277 (57.2)			314 (52.2)	997 (55.3)	
Separated	8 (1.3)	19 (0.9)			8 (1.3)	18 (1.0)	
Single	70 (11.6)	270 (12.1)			70 (11.6)	207 (11.5)	
Divorced	83 (13.8)	283 (12.7)			83 (13.8)	241 (13.4)	
Unmarried or domestic partner	0 (0.0)	2 (0.1)			0 (0.0)	0 (0.0)	
Widowed	109 (18.1)	301 (13.5)			109 (18.1)	284 (15.8)	
Unknown	17 (2.8)	79 (3.5)			17 (2.8)	56 (3.1)	
Median family income (dollar, tens)			0.001				0.029
≤5000	188 (31.3)	536 (24.0)			188 (31.3)	474 (26.3)	
>5000, ≤7000	287 (47.8)	1148 (51.5)			287 (47.8)	933 (51.7)	
>7000, ≤9000	101 (16.8)	478 (21.4)			101 (16.8)	344 (19.1)	
>9000	25 (4.2)	69 (3.1)			25 (4.2)	52 (2.9)	

**Abbreviations:** NSCLC, non-small cell lung cancer; RLN, regional lymph node.

**Table 3:** Overall survival, lung cancer-specific survival, median survival time and mean survival time in stage IIIA N0 NSCLC patients with different number of RLNs removed prior PSM

Variable	Number	5-year survival rate (%)		Median survival time (months)	Mean survival time (months)
		Overall survival	Lung cancer-specific survival		
Overall patients	11,583	6.6	8.0	11.0	21.6
Number of RLNs removed					
None	8,751	2.9	3.5	8.0	15.5
1-3	601	17.0	20.8	27.0	39.1
≥4	2,231	18.6	22.1	31.0	40.9

**Abbreviations:** NSCLC, non-small cell lung cancer; RLNs, regional lymph nodes.

**Table 4:** Univariable and multivariable Cox analyses of OS and LCSS in all patients among our cohort after PSM

Variables	Overall survival						Lung cancer-specific survival				
	Univariable analysis			Multivariable analysis			Univariable analysis			Multivariable analysis	
	HR (95% CI)	*P*		HR (95% CI)	*P*		HR (95% CI)	*P*		HR (95% CI)	*P*
Age		<0.001			<0.001			<0.001			<0.001
≤45	Reference			Reference			Reference			Reference	
45 to 54	1.112 (0.926-1.358)	0.240		1.241 (1.024-1.504)	0.028		1.086 (0.890-1.326)	0.415		1.196 (0.979-1.461)	<0.001
55 to 64	1.112 (0.936-1.346)	0.214		1.335 (1.111-1.603)	0.002		1.037 (0.858-1.253)	0.705		1.242 (1.026-1.503)	0.079
65 to 74	1.316 (1.100-1.574)	0.003		1.562 (1.302-1.873)	<0.001		1.177 (0.977-1.419)	0.086		1.413 (1.169-1.708)	0.026
≥75	1.922 (1.608-2.298)	<0.001		1.892 (1.576-2.271)	<0.001		1.705 (1.416-2.053)	<0.001		1.703 (1.408-2.059)	<0.001
Sex		<0.001			<0.001			<0.001			<0.001
Male	Reference			Reference			Reference			Reference	
Female	0.823 (0.789-0.859)			0.785 (0.751-0.822)			0.826(0.789-0.865)			0.798 (0.760-0.838)	
Race		0.001			0.001			<0.002			0.055
White	Reference			Reference			Reference			Reference	
Black	1.110 (1.043-1.182)	0.001		1.014 (0.950-1.083)	0.674		1.132 (1.058-1.211)	<0.001		1.019 (0.950-1.094)	0.600
Other	0.953 (0.878-1.036)	0.259		0.851 (0.781-0.928)	<0.001		1.018 (0.933-1.111)	0.688		0.897 (0.819-0.983)	0.020
Unknown	0.390 (0.146-1.039)	0.060		0.407 (0.152-1.090)	0.074		0.469 (0.176-1.250)	<0.130		0.515 (0.192-1.380)	0.187
Tumor size		<0.001			<0.001			<0.001			<0.001
≤1 cm	Reference			Reference			Reference			Reference	
>1, ≤2 cm	0.583 (0.146-2.332)	0.446		0.719 (0.179-2.881)	0.641		0.324 (0.048-2.428)	0.283		0.453 (0.064-3.226)	0.430
>2, ≤3 cm	0.650 (0.270-1.562)	0.335		0.663 (0.275-1.596)	0.359		0.635 (0.238-1.694)	0.365		0.663 (0.248-1.770)	0.412
>3, ≤4 cm	0.776 (0.349-1.728)	0.535		0.926 (0.415-2.071)	0.852		0.778 (0.324-1.871)	0.576		0.943 (0.391-2.275)	0.896
>4 cm	1.611 (0.403-6.442)	0.500		1.317 (0.327-5.306)	0.699		0.937 (0.132-6.653)	0.948		0.718 (0.101-5.131)	0.741
Unknown	2.226 (2.124-2.333)	<0.001		1.414 (1.336-1.495)	<0.001		2.335 (2.221-2.456)	<0.001		1.432 (1.348-1.521)	<0.001
Diagnosis year		<.001			<0.001			<0.001			<0.001
2004-2007	Reference			Reference			Reference			Reference	
2008-2011	0.812 (0.775-0.850)	<.001		0.896 (0.838-0.959)	0.002		0.793 (0.755-0.833)	<0.001		0.903 (0.839-0.972)	0.007
2012-2015	0.503 (0.470-0.538)	<.001		0.698 (0.640-0.760)	<0.001		0.476 (0.443-0.513)	<0.001		0.693 (0.631-0.761)	<0.001
Tumor location		<0.001			<0.001			<0.001			<0.001
Main bronchus	Reference			Reference			Reference			Reference	
Upper lobe	0.586 (0.532-0.644)	<0.001		0.738 (0.669-0.814)	<0.001		0.556 (0.502-0.615)	<0.001		0.718 (0.647-0.797)	<0.001
Middle lobe	0.665 (0.575-0.770)	<0.001		0.756 (0.651-0.878)	<0.001		0.628 (0.537-0.736)	<0.001		0.735 (0.625-0.863)	<0.001
Lower lobe	0.648 (0.586-0.717)	<0.001		0.772 (0.696-0.856)	<0.001		0.602 (0.541-0.670)	<0.001		0.738 (0.661-0.824)	<0.001
Overlapping lesion	0.691 (0.571-0.836)	<0.001		0.881 (0.727-1.069)	0.199		0.693 (0.566-0.848)	<0.001		0.904 (0.737-1.109)	0.335
NOS	1.244 (1.120-1.382)	<0.001		0.894 (0.799-1.002)	0.054		1.239 (1.108-1.385)	<0.001		0.890 (0.789-1.004)	0.058
Grade		<0.001			<0.001			<0.001			<0.001
Well differentiated	Reference			Reference			Reference			Reference	
Moderate differentiated	0.345 (0.310-0.385)	<0.001		0.604 (0.540-0.676)	<0.001		1.638 (1.437-1.867)	<0.001		1.463 (1.281-1.671)	<0.001
Poorly differentiated	0.532 (0.502-0.564)	<0.001		0.843 (0.792-0.898)	<0.001		2.354 (2.076-2.670)	<0.001		1.863 (1.638-2.118)	<0.001
Undifferentiated	0.745 (0.709-0.782)	<0.001		1.069 (1.015-1.126)	0.011		3.186 (2.608-3.892)	<0.001		2.344 (1.895-2.898)	<0.001
Unknown	0.978 (0.838-1.141)	0.777		1.325 (1.123-1.565)	0.001		3.174 (2.806-3.591)	<0.001		1.703 (1.500-1.933)	<0.001
Laterality		<0.001			0.675			<0.001			0.861
Left-origin of primary	Reference			Reference			Reference			Reference	
Right-origin of primary	0.959 (0.920-1.001)	0.056		0.998 (0.956-1.043)	0.941		0.942 (0.900-0.986)	0.011		0.981 (0.936-1.029)	0.436
Only one side, unspecified	2.152 (1.493-3.102)	<0.001		1.262 (0.872-1.826)	0.217		2.034 (1.361-3.039)	0.001		1.143 (0.762-1.715)	0.519
Paired site	2.233 (1.878-2.654)	<0.001		1.054 (0.880-1.262)	0.570		2.192 (1.817-2.645)	<0.001		0.994 (0.818-1.209)	0.954
Not a paired site	1.430 (0.595-3.439)	0.424		0.727(0.299-1.765)	0.480		1.678 (0.698-4.034)	0.248		0.806 (0.331-1.960)	0.634
Histologic type		<0.001			<0.001			<0.001			<0.001
Adenocarcinoma	Reference			Reference			Reference			Reference	
Squamous cell carcinoma	1.179 (1.122-1.238)	<0.001		1.192 (1.130-1.258)	<0.001		1.148 (1.088-1.212)	<0.001		1.178 (1.111-1.249)	<0.001
Adenosquamous	1.065 (0.885-1.281)	0.507		1.355 (1.124-1.634)	0.001		1.098 (0.901-1.338)	0.353		1.420 (1.164-1.734)	0.001
Large cell carcinoma	1.428 (1.265-1.612)	<0.001		1.283 (1.125-1.463)	<0.001		1.431 (1.255-1.632)	<0.001		1.265 (1.097-1.457)	0.001
Other types of NSCLC	1.055 (0.999-1.114)	0.053		1.019 (0.963-1.078)	0.515		1.065 (1.004-1.130)	0.035		1.036 (0.975-1.100)	0.259
RLNs removed		<0.001			<0.001			<0.001			<0.001
With RNLs removed	Reference			Reference			Reference			Reference	
Without any RLN removed	0.275 (0.259-0.291)			0.284 (0.265-0.305)			0.239 (0.224-0.256)			0.252 (0.233-0.273)	
Radiotherapy record		<0.001			<0.001			<0.001			<0.001
Beam radiation	Reference			Reference			Reference			Reference	
Beam with implants or isotopes	1.401 (0.829-2.368)	0.208		1.520 (0.888-2.602)	0.127		1.545 (0.896-2.665)	0.118		1.673 (0.956-2.926)	0.071
Radiation, but not specified	1.004 (0.788-1.279)	0.976		0.911 (0.714-1.163)	0.455		1.004 (0.772-1.305)	0.978		0.907 (0.696-1.181)	0.469
Radioactive implants	0.865 (0.574-1.304)	0.488		0.774 (0.502-1.193)	0.246		0.661 (0.398-1.098)	0.110		0.629 (0.374-1.059)	0.081
Recommended, unknown	1.043 (0.804-1.352)	0.752		1.081 (0.832-1.404)	0.560		1.130 (0.863-1.480)	0.374		1.211 (0.923-1.589)	0.166
Refused	1.797 (1.524-2.120)	<0.001		1.154 (0.975-1.367)	0.096		1.880 (1.581-2.237)	<0.001		1.246 (1.043-1.490)	0.015
None/Unknown	1.095 (1.048-1.145)	<0.001		1.332 (1.260-1.409)	<0.001		1.085 (1.034-1.138)	0.001		1.345 (1.267-1.428)	<0.001
Radiation sequence		<0.001			<0.001			<0.001			<0.001
Prior to surgery	Reference			Reference			Reference			Reference	
After surgery	1.385 (1.132-1.696)	0.002		1.095 (0.893-1.343)	0.384		1.444 (1.151-1.812)	0.002		1.140 (0.907-1.434)	0.262
Before and after surgery	1.006 (0.589-1.719)	0.983		0.735 (0.427-1.267)	0.268		1.194 (0.682-2.091)	0.535		0.832 (0.471-1.471)	0.527
Intraoperative radiation	3.062 (0.428-21.911)	0.265		2.616(0.344-19.890)	0.353		0.005 (0.000-1.558E+)	0.843		0.103(0.000-4.858E+)	0.814
Unknown, but both given	1.358 (0.432-4.268)	0.601		0.413 (0.132-1.420)	0.167		1.128 (0.278-4.575)	0.866		0.388 (0.092-1.647)	0.199
No radiation and/or surgery	1.883 (0.599-5.920)	0.279		1.143 (0.363-3.604)	0.819		2.362 (0.749-7.452)	0.143		1.342 (0.424-4.249)	0.617
In and before/after surgery	2.122 (1.777-2.534)	<0.001		0.693 (0.575-0.836)	<0.001		2.271 (1.859-2.775)	<0.001		0.720 (0.583-0.888)	0.002
Chemotherapy record											
No	Reference			Reference			Reference			Reference	
Yes	0.714 (0.684-0.745)	<0.001		0.655 (0.625-0.686)	<0.001		0.778 (0.743-0.814)	<0.001		0.704 (0.670-0.741)	<0.001
Marital status		<0.001			<0.001			<0.001			<0.001
Married	Reference			Reference			Reference			Reference	
Separated	1.121 (0.917-1.371)	0.266		1.123 (0.917-1.376)	0.263		1.181 (0.956-1.459)	0.123		1.175 (0.949-1.456)	0.138
Single	1.198 (1.112-1.278)	<0.001		1.184 (1.106-1.267)	<0.001		1.165 (1.086-1.251)	<0.001		1.144 (1.062-1.232)	<0.001
Divorced	1.060 (0.991-1.135)	0.091		1.126 (1.050-1.208)	0.001		1.059 (0.984-1.140)	0.125		1.124 (1.043-1.213)	0.002
Unmarried or domestic partner	0.671 (0.216-2.082)	0.490		0.559 (0.180-1.739)	0.315		0.770 (0.248-2.388)	0.651		0.691 (0.222-2.150)	0.523
Widowed	1.387 (1.315-1.462)	<0.001		1.145 (1.080-1.215)	<0.001		1.345 (1.269-1.425)	<0.001		1.124 (1.054-1.198)	<0.001
Unknown	1.051 (0.933-1.183)	0.414		1.029 (0.912-1.161)	0.642		1.011 (0.887-1.151)	0.875		0.987 (0.864-1.127)	0.846
Median family income (dollar, tens)		<0.001			0.002			<0.001			0.548
≤5000	Reference			Reference			Reference			Reference	
>5000, ≤7000	0.857 (0.817-0.899)	<0.001		0.938 (0.891-0.986)	0.013		0.852 (0.809-.0897)	<0.001		0.936 (0.886-0.989)	0.019
>7000, ≤9000	0.806 (0.758-0.857)	<0.001		0.891 (0,831-0.954)	0.001		0.813 (0.761-0.869)	<0.001		0.896 (0.832-0.966)	0.004
>9000	0.806 (0.713-0.912)	0.001		0.840 (0.739-0.956)	0.008		0.792 (0.692-0.907)	0.001		0.826 (0.718-0.951)	0.008

**Abbreviations:** OS, overall survival; LCSS, lung cancer-specific survival; NSCLC, non-small cell lung cancer; HR, hazard ratio; CI, confidence interval; RLN, regional lymph node.

**Table 5:** Univariable and multivariable Cox analyses of OS and LCSS in patients with RLNs removed after PSM

Variables	Overall survival						Lung cancer-specific survival				
	Univariable analysis			Multivariable analysis			Univariable analysis			Multivariable analysis	
	HR (95% CI)	*P*		HR (95% CI)	*P*		HR (95% CI)	*P*		HR (95% CI)	*P*
Age		<0.001			<0.001			0.001			<0.001
≤45	Reference			Reference			Reference			Reference	
45 to 54	1.525 (0.902-2.578)	0.116		1.667 (0.981-2.832)	0.059		1.214 (0.712-2.070)	0.476		1.363 (0.795-2.338)	0.260
55 to 64	1.347 (0.814-2.230)	0.247		1.426 (0.857-2.373)	0.172		0.990 (0.595-1.647)	0.969		1.088 (0.650-1.821)	0.748
65 to 74	1.674 (1.017-2.756)	0.043		1.935 (1.167-3.208)	0.010		1.261 (0.764-2.082)	0.364		1.508 (0.907-2.510)	0.114
≥75	2.233 (1.353-3.684)	0.002		2.909 (1.750-4.838)	<0.001		1.476 (0.890-2.447)	0.131		2.002 (1.196-3.353)	0.008
Sex		<0.001			<0.001			<0.001			<0.001
Male	Reference			Reference			Reference			Reference	
Female	0.574 (0.512-0.644)			0.618 (0.548-0.697)			0.565 (0.495-0.646)			0.627 (0.545-0.721)	
Race		0.480						0.860			
White	Reference						Reference				
Black	1.034 (0.853-1.254)	0.734					1.087 (0.873-1.354)	0.456			
Other	0.836 (0.645-1.085)	0.178					1.033 (0.784-1.362)	0.816			
Unknown	0.503 (0.071-3.571)	0.492					0.669 (0.094-4.754)	0.688			
Tumor size		<0.001			0.048			<0.001			0.069
≤1 cm	Reference			Reference			Reference			Reference	
>1, ≤2 cm	0.000 (0.000-2.900E+)	0.934		0.000 (0.000-5.934E+)	0.945		0.000 (0.000-6.954E+)	0.942		0.000 (0.000-2.382E+)	0.954
>2, ≤3 cm	0.000 (0.000-1.615E+)	0.883		0.000 (0.000-6.192E+)	0.892		0.000 (0.000-1.346E+)	0.903		0.000 (0.000-2.732E+)	0.923
>3, ≤4 cm	0.425 (0.060-3.019)	0.392		0.346 (0.048-2.500)	0.293		0.589 (0.083-4.184)	0.596		0.467 (0.064-3.384)	0.451
Unknown	1.740 (1.362-2.222)	<0.001		1.468 (1.133-1.902)	0.004		1.953 (1.490-2.559)	<0.001		1.522 (1.140-2.031)	0.004
Diagnosis year		<0.001			0.003			<0.001			0.017
2004-2007	Reference			Reference			Reference			Reference	
2008-2011	0.881 (0.778-0.999)	0.048		0.933 (0.771-1.131)	0.481		0.857 (0.742-0.990)	0.035		0.934 (0.750-1.165)	0.546
2012-2015	0.636 (0.529-0.765)	<0.001		0.696 (0.549-0.881)	0.003		0.614 (0.499-0.756)	<0.001		0.706 (0.540-0.923)	0.011
Tumor location		<0.001			<0.001			<0.001			<0.001
Main bronchus	Reference			Reference			Reference			Reference	
Upper lobe	0.401 (0.283-0.568)	<0.001		0.490 (0.342-0.701)	<0.001		0.366 (0.249-0.539)	<0.001		0.446 (0.299-0.664)	<0.001
Middle lobe	0.496 (0.316-0.777)	0.002		0.565 (0.356-0.897)	0.016		0.403 (0.240-0.678)	0.001		0.471 (0.276-0.805)	0.006
Lower lobe	0.447 (0.313-0.638)	<0.001		0.585 (0.406-0.844)	0.004		0.413 (0.278-0.613)	<0.001		0.550 (0.366-0.827)	0.004
Overlapping lesion	0.746 (0.424-1.311)	0.308		0.733 (0.409-1.314)	0.297		0.727 (0.387-1.367)	0.322		0.714 (0.372-1.374)	0.313
NOS	0.425 (0.257-0.703)	0.001		0.614 (0.367-1.027)	0.063		0.396 (0.224-0.701)	0.001		0.569 (0.317-1.022)	0.059
Grade		<0.001			<0.001			<0.001			<0.001
Well differentiated	Reference			Reference			Reference			Reference	
Moderate differentiated	1.427 (1.160-1.754)	0.001		1.314 (1.061-1.628)	0.012		1.522 (1.186-1.954)	0.001		1.400 (1.081-1.812)	0.011
Poorly differentiated	2.145 (1.757-2.619)	<0.001		1.728 (1.397-2.137)	<0.001		2.377 (1.868-3.025)	<0.001		1.869 (1.447-2.414)	<0.001
Undifferentiated	2.847 (1.953-4.151)	<0.001		2.861 (1.800-4.549)	<0.001		3.379 (2.197-5.197)	<0.001		3.477 (2.078-5.818)	<0.001
Unknown	1.815 (1.441-2.287)	<.001		1.383 (1.085-1.764)	0.009		2.189 (1.667-2.875)	<0.001		1.588 (1.193-2.114)	0.002
Laterality		0.120						0.163			
Left-origin of primary	Reference						Reference				
Right-origin of primary	0.888 (0.793-0.995)	0.040					0.884 (0.775-1.007)	0.064			
Paired site	1.148 (0.161-8.167)	0.890					1.478 (0.208-10.518)	0.697			
Histologic type		<0.001			<0.001			<0.001			<0.001
Adenocarcinoma	Reference			Reference			Reference			Reference	
Squamous cell carcinoma	1.738 (1.521-1.986)	<0.001		1.231 (1.065-1.422)	0.005		1.790 (1.531-2.092)	<0.001		1.220 (1.030-1.445)	0.021
Adenosquamous	1.898 (1.388-2.595)	<0.001		1.644 (1.192-2.267)	0.002		2.347 (1.677-3.284)	<0.001		1.959 (1.385-2.772)	<0.001
Large cell carcinoma	1.813 (1.297-2.536)	0.001		0.837 (0.544-1.287)	0.417		1.793 (1.203-2.674)	0.004		0.723 (0.439-1.191)	0.203
Other types of NSCLC	0.969 (0.835-1.126)	0.685		0.957 (0.816-1.123)	0.591		1.062 (0.894-1.262)	0.494		1.053 (0.877-1.265)	0.581
RLNs removed		0.003			0.088			0.004			0.179
≥4 RLNs removed	Reference			Reference			Reference			Reference	
1-3 RLNs removed	1.212 (1.069-1.347)			1.118 (0.983-1.271)			1.234 (1.068-1.426)			1.107 (0.954-1.284)	
Radiotherapy record		<0.001			0.304			<0.001			0.022
Beam radiation	Reference			Reference			Reference			Reference	
Radiation, but not specified	0.846 (0.400-1.787)	0.661		0.770 (0.358-1.657)	0.504		0.916 (0.408-2.054)	0.831		0.845 (0.369-1.937)	0.691
Radioactive implants	0.740 (0.276-1.983)	0.550		0.429 (0.136-1.355)	0.149		0.435 (0.108-1.749)	0.241		0.352 (0.086-1.436)	0.145
Recommended, unknown	1.079 (0.593-1.966)	0.803		0.996 (0.519-1.911)	0.991		1.316 (0.721-2.401)	0.371		1.265 (0.648-2.470)	0.492
Refused	1.611 (0.667-3.895)	0.289		1.618 (0.651-4.024)	0.300		1.920 (0.794-4.647)	0.148		1.928 (0.762-4.878)	0.166
None/Unknown	0.584 (0.517-0.660)	<0.001		0.850 (0.670-1.079)	0.182		0.506 (0.441-0.581)	<0.001		0.732 (0.551-0.973)	0.032
Radiation sequence		<0.001			0.001			<0.001			0.004
Prior to surgery	Reference			Reference			Reference			Reference	
After surgery	1.503 (1.186-1.906)	0.001		1.387 (1.082-1.779)	0.010		1.489 (1.143-1.940)	0.003		1.393 (1.056-1.838)	0.019
Before and after surgery	1.147 (0.597-2.204)	0.681		1.695 (0.867-3.314)	0.123		1.416 (0.731-2.745)	0.302		1.984 (1.004-3.922)	0.049
Intraoperative radiation	4.900 (0.682-35.225)	0.114		22.610 (1.839-278.002)	0.015		0.001 (0.000-1.284E+)	0.922		0.000 (0.000-8.411E+)	0.979
Unknown, but both given	7.228 (2.278-22.931)	0.001		5.759 (1.755-18.896)	0.004		8.550 (2.680-27.280)	<0.001		7.453 (2.244-24.756)	0.001
No radiation and/or surgery	0.800 (0.643-0.994)	0.044					0.701 (0.549-0.895)	0.004			
Chemotherapy record		0.834						0.009			0.407
No	Reference						Reference			Reference	
Yes	1.012 (0.903-1.135)	0.002					1.192 (1.046-1.358)	0.001		0.934 (0.794-1.098)	0.001
Marital status		0.490						0.851			
Married	Reference						Reference				
Separated	0.987 (0.581-1.675)	0.960					1.114 (0.628-1.974)	0.712			
Single	1.014 (0.843-1.220)	0.884					0.948 (0.762-1.179)	0.631			
Divorced	1.041 (0.879-1.233)	0.644					1.035 (0.852-1.257)	0.730			
Widowed	1.110 (0.949-1.298)	0.190					1.003 (0.833-1.209)	0.971			
Unknown	0.739 (0.496-1.101)	0.137					0.764 (0.489-1.105)	0.239			
Median family income (dollar, tens)		0.105						0.133			
≤5000	Reference						Reference				
>5000, ≤7000	0.914 (0.802-1.042)	0.179					0.937 (0.804-1.093)	0.409			
>7000, ≤9000	0.812 (0.685-0.962)	0.016					0.884 (0.727-1.075)	0.217			
>9000	1.007 (0.728-1.391)	0.968					1.183 (0.831-1.685)	0.351			

**Abbreviations:** OS, overall survival; LCSS, lung cancer-specific survival; NSCLC, non-small cell lung cancer; HR, hazard ratio; CI, confidence interval; RLN, regional lymph node.

## Abbreviations

LC: lung cancer
NSCLC: non-small cell lung cancer
RLN: regional lymph node
SEER: Surveillance, Epidemiology and End Results
LCSS: lung cancer-specific survival
OS: overall survival
PSM: propensity score method
RT: radiotherapy
CT: chemotherapy
HR: hazard ratio
CI: confidence interval
MDST: median survival time
MST: mean survival time
