# Supplementary material for: The Valuable Prognostic Impact of Regional Lymph Node Removed on Outcomes for IIIA N0 NSCLC Patients
Source: J Cancer. 2023 Jul 9;14(11):2093–108. doi: 10.7150/jca.86495 (PMC10367914; doi:10.7150/jca.86495)
Supplement: Supplementary file 1 — Supplementary figures. [file jcav14p2093s1.pdf]

Figure S1 Comparison of survival curve: role of CT and RT on survival outcome in all patients among our cohort

(A) CT that can influence OS in all patients among our cohort;
(B) CT that can influence LCSS in all patients among our cohort;
(C) RT that can influence OS in all patients among our cohort;
(D) RT that can influence LCSS in all patients among our cohort.

Abbreviations: HR, hazard ratio; CI, confidence interval.

Figure S2 Comparison of survival curve: role of CT and RT on survival outcome in patients with RLNs removed among our cohort

(A) CT that can influence OS in patients with RLNs removed among our cohort;
(B) CT that can influence LCSS in patients with RLNs removed among our cohort;
(C) RT that can influence OS in patients with RLNs removed among our cohort;
(D) RT that can influence LCSS in patients with RLNs removed among our cohort.

Abbreviations: HR, hazard ratio; CI, confidence interval.

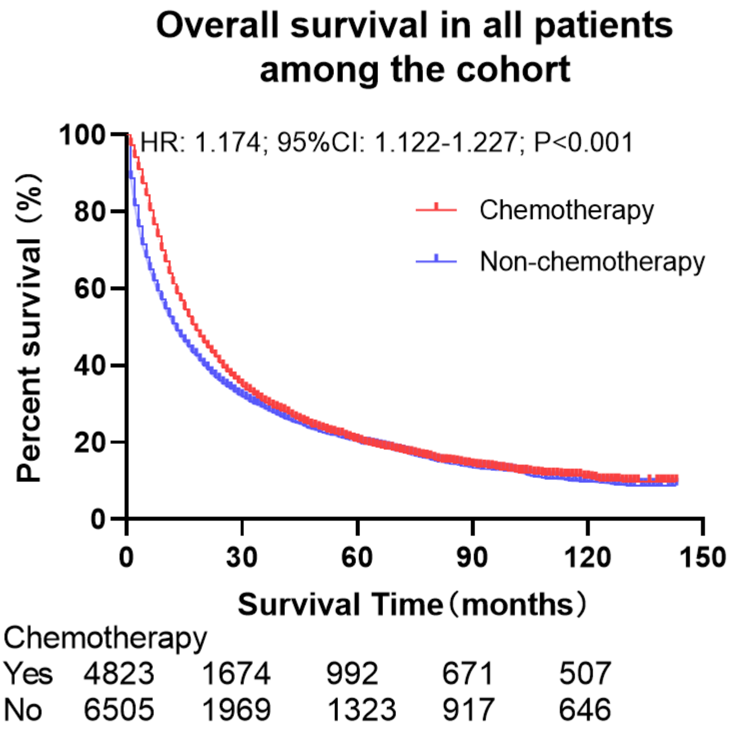
Overall survival in all patients among the cohort
HR: 1.174; 95%CI: 1.122-1.227; P<0.001
Chemotherapy
Non-chemotherapy
Percent survival (%)
100
80
60
40
20
0
0
30
60
90
120
150
Survival Time (months)
Chemotherapy
Yes 4823 1674 992 671 507
No 6505 1969 1323 917 646

Figure S1A

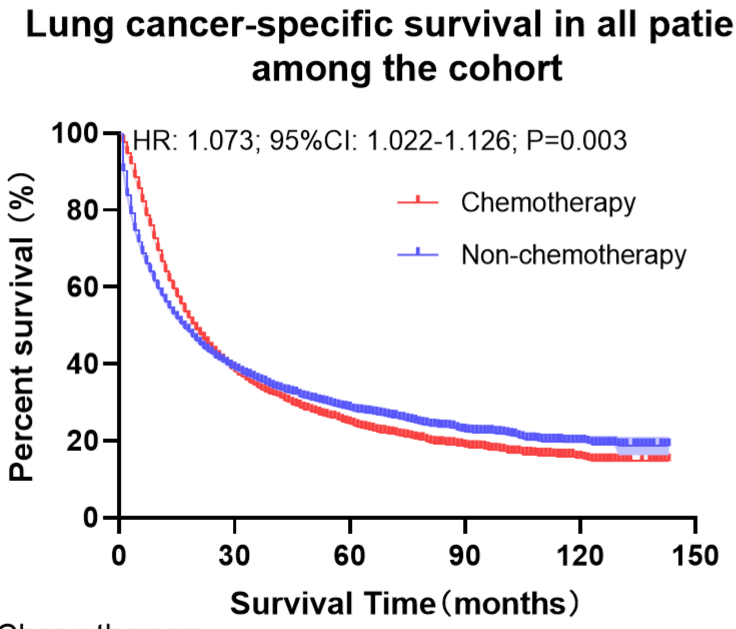

Chemotherapy					
Yes	4823	1838	1192	888	735
No	6505	2492	1848	1478	1279

Figure S1B

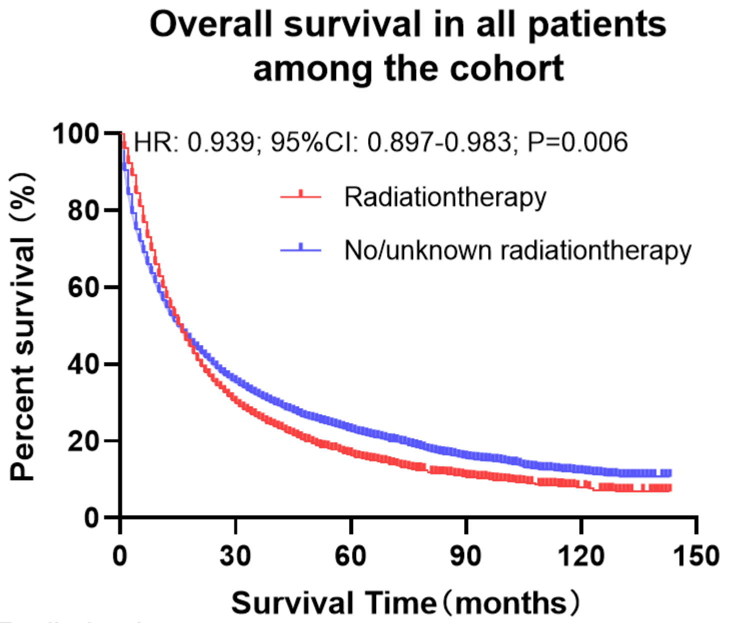

Radiationtherapy					
Yes	4013	1195	1068	421	313
No	7225	2579	1604	1156	831

Figure S1C

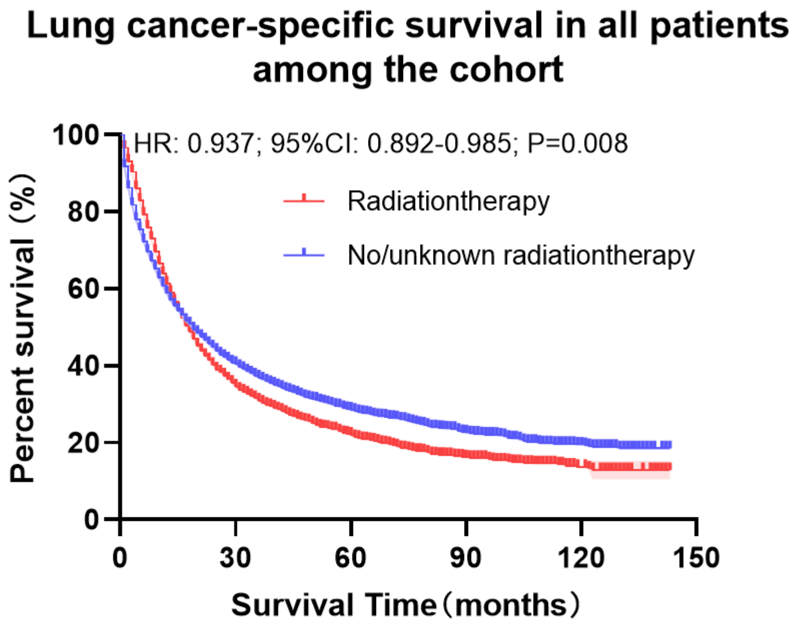

Radiationtherapy					
Yes	4013	1389	897	642	546
No	7225	2951	2023	1638	1416

Figure S1D

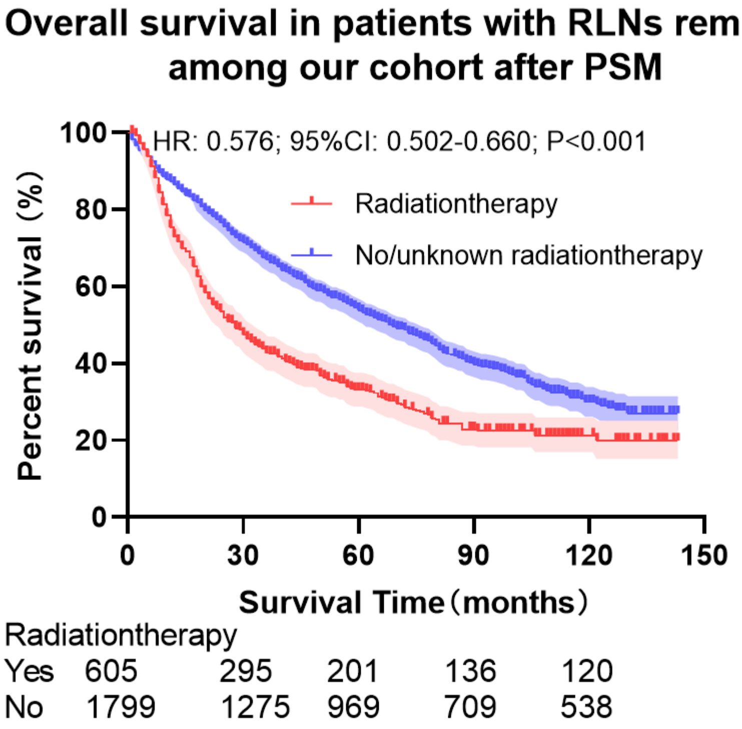
Overall survival in patients with RLNs removed among our cohort after PSM
HR: 0.576; 95%CI: 0.502-0.660; P<0.001
Radiationtherapy
No/unknown radiationtherapy
Percent survival (%)
100
80
60
40
20
0
0
30
60
90
120
150
Survival Time（months）
Radiationtherapy
Yes 605 295 201 136 120
No 1799 1275 969 709 538

Figure S2A

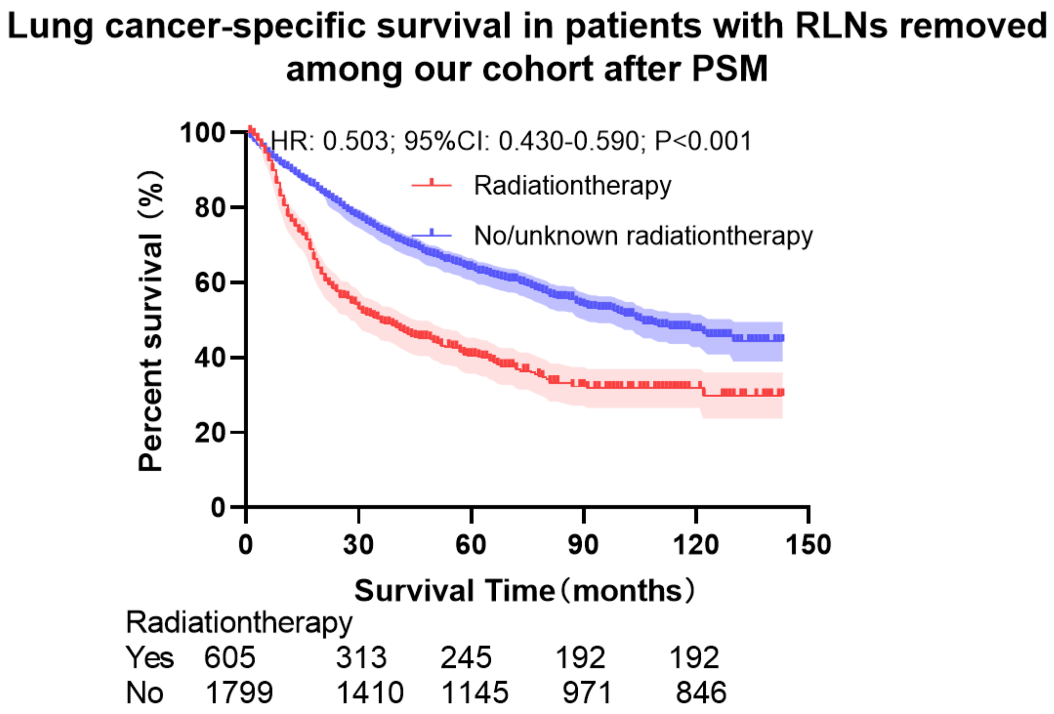
Lung cancer-specific survival in patients with RLNs removed among our cohort after PSM
HR: 0.503; 95%CI: 0.430-0.590; P<0.001
Radiationtherapy
No/unknown radiationtherapy
Percent survival（%）
100
80
60
40
20
0
0
30
60
90
120
150
Survival Time（months）
Radiationtherapy
Yes 605 313 245 192 192
No 1799 1410 1145 971 846

Figure S2B

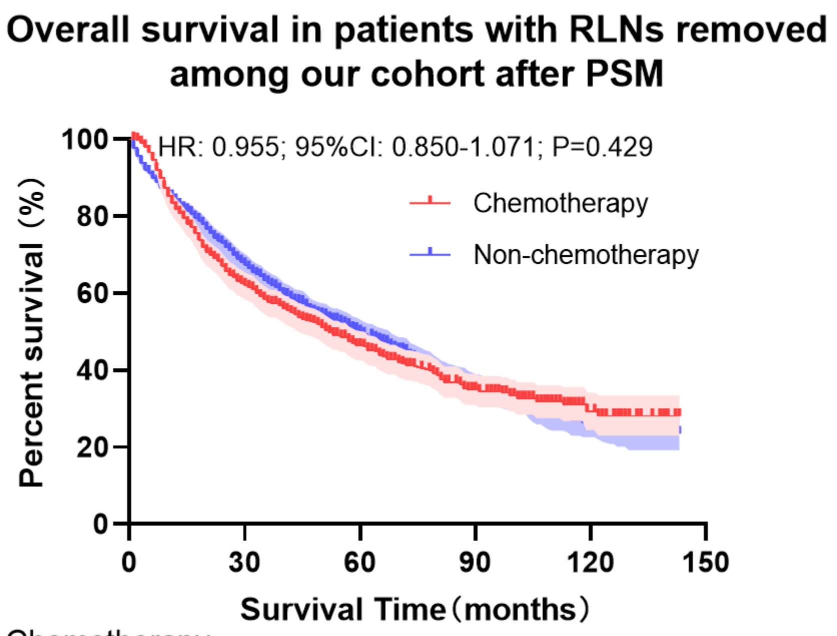

Chemotherapy					
Yes	949	646	438	332	296
No	1455	968	735	522	386

Figure S2C

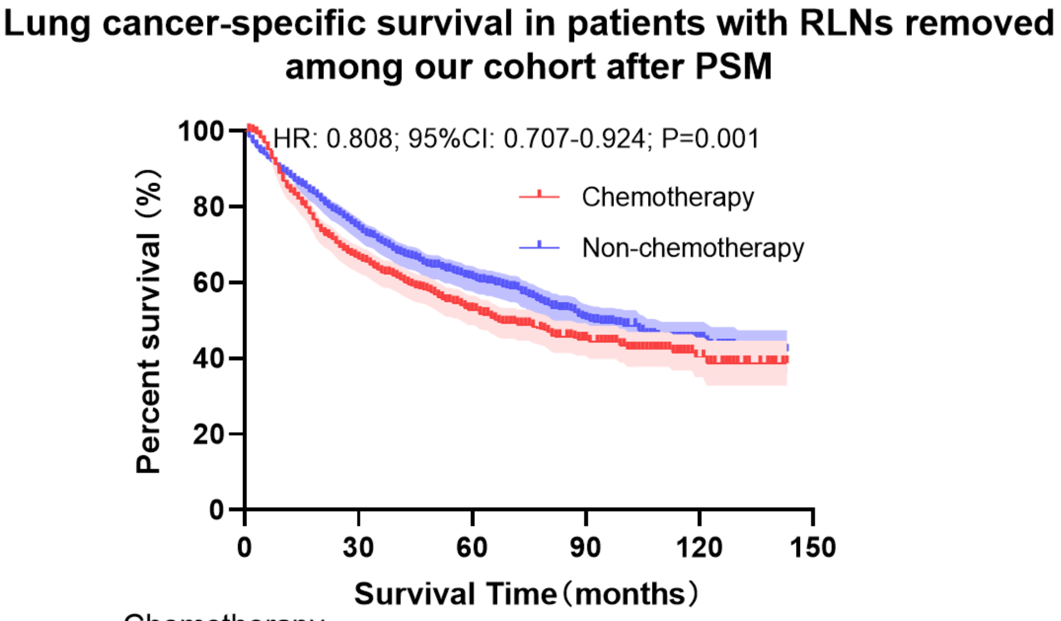

Chemotherapy

Yes	949	628	498	427	365
No	1455	1079	892	736	655

Figure S2D
